# LINC01764 promotes colorectal cancer cells proliferation, metastasis, and 5‐fluorouracil resistance by regulating glucose and glutamine metabolism via promoting c‐MYC translation

**DOI:** 10.1002/mco2.70003

**Published:** 2024-11-11

**Authors:** Ran Duan, Yujia Zhai, Qiushuang Wang, Liqin Zhao, Yixuan Wang, Nuoya Yu, Jieyun Zhang, Weijian Guo

**Affiliations:** ^1^ Department of Gastrointestinal Medical Oncology Fudan University Shanghai Cancer Center Shanghai China; ^2^ Department of Oncology Shanghai Medical College Fudan University Shanghai China; ^3^ Department of Medical Oncology Fujian Cancer Hospital and Fujian Medical University Cancer Hospital Fujian Medical University Fuzhou China; ^4^ Department of Oncology Ruijin Hospital Shanghai Jiao Tong University School of Medicine Shanghai China

**Keywords:** 5‐FU resistance, CRC, lncRNA, metastasis, proliferation

## Abstract

Few biomarkers are available for predicting chemotherapeutic response and prognosis in colorectal cancer (CRC). Long‐noncoding RNAs (lncRNAs) are essential for CRC development and growth. Therefore, studying lncRNAs may reveal potential predictors of chemotherapy response and prognosis in CRC. LINC01764 was analyzed using datasets from Fudan University Shanghai Cancer Center's advanced CRC patients’ RNA‐seq and The Cancer Genome Atlas datasets. Gene set enrichment analysis was employed to detect related pathways. Cotransfection experiments, RNA pulldown assays, RNA‐binding protein immunoprecipitation, protein synthesis activity, and dual‐luciferase reporter assays were performed to determine interactions among LINC01764, hnRNPK, and c‐MYC. High LINC01764 expression correlates with metastasis, a poor response to FOLFOX/XELOX chemotherapy, and a poor prognosis in CRC. LINC01764 enhance glycolysis and glutamine metabolism to promote CRC cells proliferation, metastasis, and 5‐fluorouracil (5‐FU) resistance. LINC01764 specifically binds to hnRNPK, facilitating its interaction with c‐MYC mRNA and promoting internal ribosome entry site (IRES)‐dependent translation of c‐MYC, thereby exerting oncogenic effects. LINC01764 induced 5‐FU chemoresistance by upregulating the c‐MYC, glucose, and glutamine metabolism pathways, which downregulated *UPP1*, crucial for activating 5‐FU. Conclusively, LINC01764 promotes CRC progression and 5‐FU resistance through hnRNPK‐mediated‐c‐MYC IRES‐dependent translational regulation, which suggests its potential as a predictor of CRC chemotherapy response and prognosis.

## Graphical abstract

LINC01764 enhanced glycolysis and glutamine metabolism to promote colorectal cells progression and 5‐FU resistance. LINC01764 specifically binds to hnRNPK and promotes its binding on c‐MYC mRNA, which could promote IRES‐dependent translation of c‐MYC and thus performs its oncogenic functions. LINC01764 induces 5‐FU chemoresistance by promoting the c‐MYC, glucose, and glutamine metabolism pathways to downregulate UPP1 mRNA expression, which facilitates the conversion of 5‐FU to active ingredient.

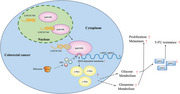

## 1 INTRODUCTION

Colorectal cancer (CRC) is a prevalent gastrointestinal cancer with high morbidity and mortality rates, ranking fifth worldwide in terms of newly diagnosed cancer cases and cancer‐related deaths, causing approximately 600,000 deaths in 2020.^1^ Despite advancements in CRC diagnosis and screening technologies, approximately 20% of patients are diagnosed with distant metastasis during initial diagnosis, resulting in poor prognosis and shortened survival.^2^ Over the past few decades, major breakthroughs have been made in CRC treatment, including endoscopic therapy, local surgical excision, preoperative radiotherapy, chemotherapy, targeted therapy, and immunotherapy, which have prolonged the overall survival (OS) of patients with advanced CRC by 3 years. Immunotherapy has been extensively developed and is particularly effective for deficient mismatch repair/microsatellite instability‐high CRC, showing greater benefits than chemotherapy. However, approximately 90% of CRC cases are proficient mismatch repair/microsatellite stable CRC, rendering immunotherapy ineffective.^3^ Therefore, chemotherapy, often combined with or without molecular‐targeted therapy, remains the cornerstone of treatment.^4^, ^5^ While initially beneficial for most patients, chemoresistance inevitably develops.^6^ Therefore, identifying key regulatory molecules of CRC metastasis and chemoresistance is not only significant for exploring the underlying mechanism but also for predicting chemotherapy response.

Long noncoding RNAs (lncRNAs), which are nonprotein‐coding transcripts with lengths > 200 nucleotides, constitute 68% of the human transcriptome and play significant roles in various physiological processes. They are also implicated in the pathophysiology of several diseases, including neurological diseases, cardiovascular diseases, and cancer.^7^, ^8^, ^9^, ^10^ Abnormal lncRNA expression and mutation are associated with the onset and progression of several cancers, including colon, liver, breast, lung, bladder cancer, and leukemia.^11^ LncRNAs can function as either tumor suppressors or promoters.^12^ In colon cancer, they participate in various pathophysiological processes, including the epithelial–mesenchymal transition (EMT), chemoresistance, cell stemness, apoptosis, and cellular energy metabolism.^13^, ^14^, ^15^ For example, TUG1 inhibits EMT, whereas CYTOR promotes it. CCAT1 is associated with 5‐fluorouracil (5‐FU) resistance.^14^ LINC01554 regulates tumor metabolism by affecting the stability of PKM2 through the ubiquitin–proteasome pathway.^15^ LncRNAs function through various mechanisms, including interactions with RNA, DNA, and proteins in the nucleus, and through four cytoplasmic modes of action—signaling, decoy, guide, and scaffold.^16^ For example, H19 sponges miR‐138 and miR‐200a to regulate the expression of vimentin, ZEB1, and ZEB2 in colon cancer cells.^17^ MALAT1 is upregulated in colon cancer and interacts with splicing regulator SR protein family members.^18^ Despite intensive study of lncRNAs, there are few clinically applicable lncRNAs for CRC that can predict both chemotherapy efficacy and patient prognosis.

In this study, we focused on the upregulated lncRNAs in colon cancer based on analyses of datasets from the Fudan University Shanghai Cancer Center (FDUSCC) and The Cancer Genome Atlas (TCGA). We characterized a novel oncogenic lncRNA, LINC01764, which is located on chromosome 19, at region p13.12, spanning 381 bp. LINC01764 is located on the antisense strand of UCA1, another lncRNA. Few studies have reported the effect of LINC01764 on tumors, except for one study that demonstrated that LINC01764 expression was relatively low in bladder cancer tissue, and its low expression was associated with a poor prognosis. In contrast, our findings indicate increased expression of LINC01764 in tumor tissues where it promotes CRC progression and 5‐FU resistance. Subsequently, we investigated the function and action mechanism of LINC01764 in CRC and explored its potential clinical value as a predictor of the response to chemotherapy.

## 2 RESULTS

### 2.1 Discovery of oncogenic lncRNAs and characterization of LINC01764 in CRC

**FIGURE 1:**
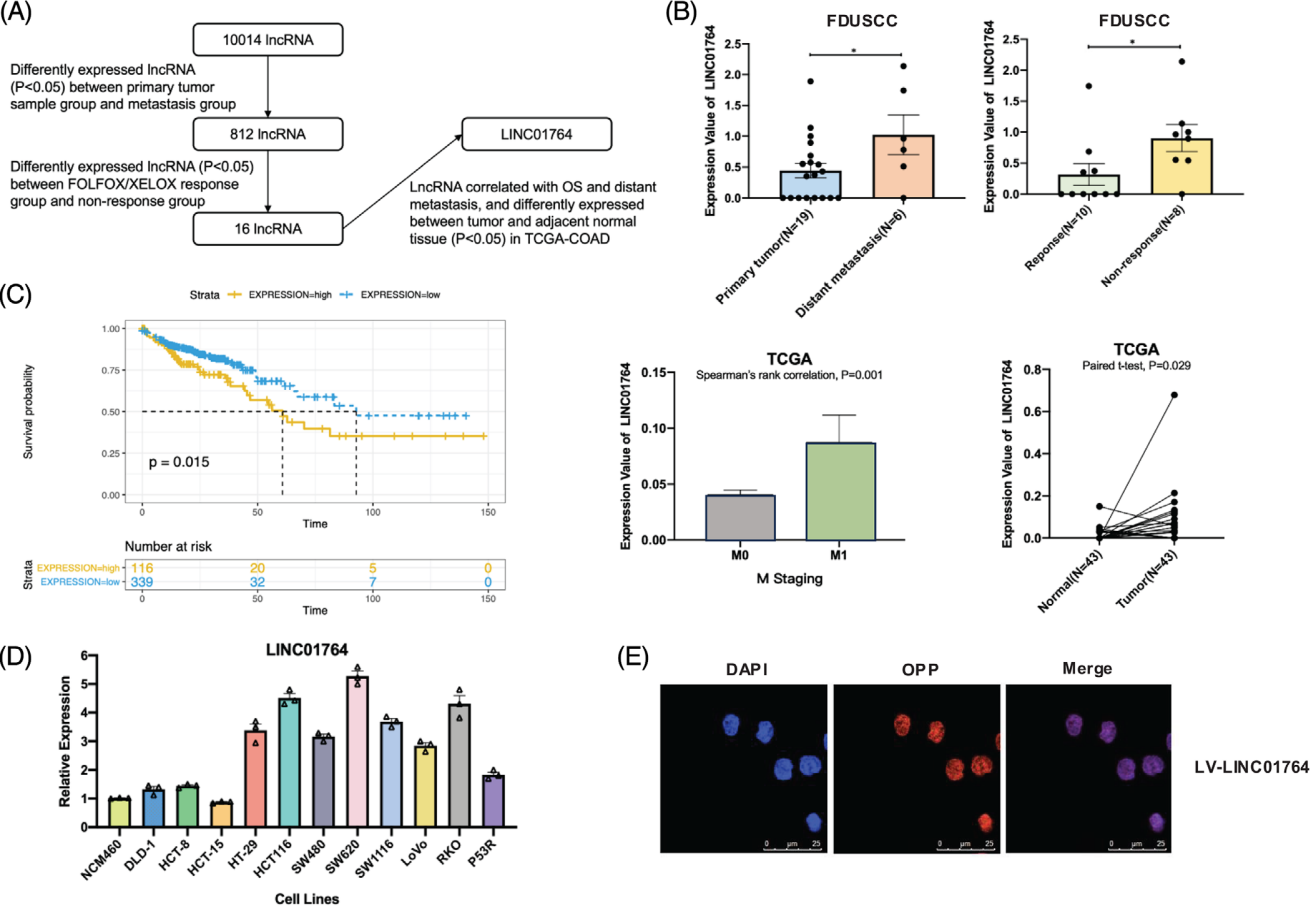
Discovery of oncogenic lncRNAs and characterization of LINC01764 in CRC. (A) Flowchart showing the process of screening oncogenic lncRNA in CRC. (B) In FUDSCC dataset, expression of LINC01764 was significantly higher in metastasis sample group and nonresponse group. (C) In TCGA‐COAD dataset, patients with high expression of LINC01764 turned out significantly shorter OS, M1 stage patients have higher LINC01764 expression, the expression in cancer tissue is significantly higher than that in adjacent normal tissue. (D) Expression of LINC01764 in human colon epithelial cell NCM460 and 11 CRC cell lines, HT‐29, HCT116, SW480, SW620, SW1116, LoVo, and RKO has significantly higher expression than NCM460. (E) FISH was conducted to detect and visualize the distribution of LINC01764 in CRC cells. Red fluorescence (LINC01764‐labeled probe) indicates LINC01764. Nuclei were stained with DAPI (blue). All data are shown as the mean ± SEM values. *p < 0.05.

To identify potential lncRNAs involved in CRC progression and chemoresistance, we performed high‐throughput RNA sequencing of 25 tissue samples from CRC patients with distant metastasis at FDUSCC. The samples were divided into two groups—primary tumor samples (colonoscopy biopsy specimens; *N* = 19) and metastasis samples (distant metastasis needle biopsy specimens; *N* = 6). These groups were unpaired samples from different patients, and 812 differentially expressed lncRNAs between the two groups were identified. In the second step, 18 CRC patients treated with FOLFOX/XELOX were divided into the response (*N* = 10) and nonresponse (*N* = 8) groups based on the Response Evaluation Criteria in Solid Tumors. A total of 323 differentially expressed lncRNAs between these groups were identified, 16 of which overlapped with the initial screening results. Subsequently, the Colon Adenocarcinoma (COAD) dataset of TCGA database was utilized to analyze the correlation of the 16 candidate lncRNAs with OS and distant metastasis and compared the differences in the expression of these lncRNAs in cancer tissue and adjacent normal tissue. Patients with high LINC01764 expression had reduced survival times, those with distant metastasis exhibited increased LINC01764 expression, and the expression in cancer tissues was significantly higher than that in adjacent normal tissues (Figure 1A–C). Consistently, we observed higher LINC01764 expression in several CRC cell lines than in the human colon epithelial cell line NCM460 (Figure 1D). Furthermore, fluorescence in situ hybridization (FISH) assays demonstrated that LINC01764 was mainly localized within the nuclei (Figure 1E). These results indicate the potential oncogenic characteristics of LINC01764 and provide evidence for further study.

### 2.2 LINC01764 promotes the proliferation, migration, stemness, and 5‐FU resistance of CRC cells in vitro

**FIGURE 2:**
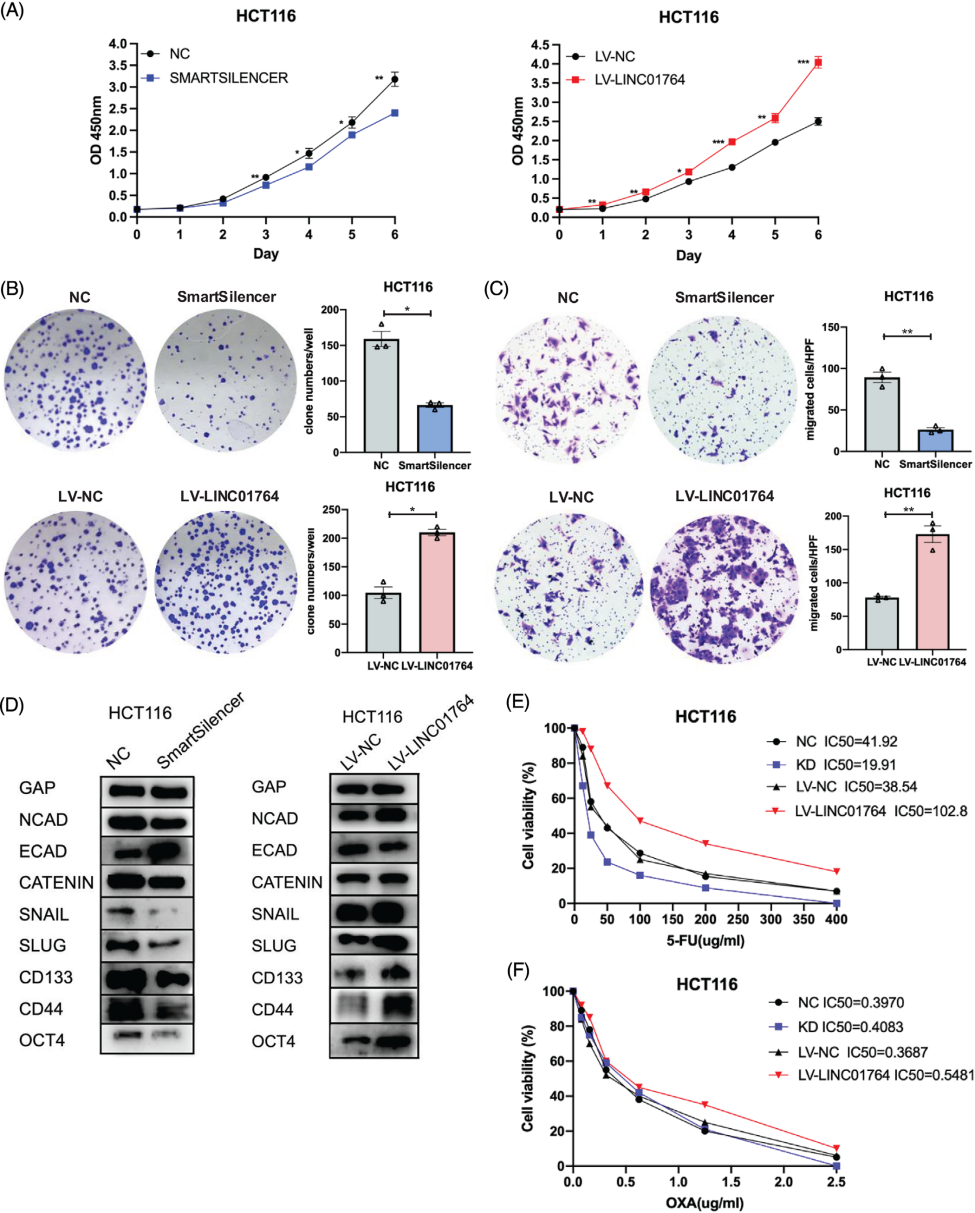
LINC01764 facilitates the proliferation and migration of CRC cells and weakens its sensitivity to 5‐FU in vitro. (A and B) Cell proliferation was assessed by CCK‐8 (A) and colony formation (B). Downregulation of LINC01764 markedly inhibited the proliferation of HCT116 cells while upregulation of LINC01764 promoted proliferation. (C) Cell migration was assessed by Transwell assays. Downregulation of LINC01764 significantly inhibited the migration of HCT116 while upregulation of LINC01764 promoted migration. (D) Western blot analysis was performed to assess the protein levels of N‐cadherin, E‐cadherin, β‐catenin, Snail, Slug, CD133, CD44, and Oct4 after knocking down or overexpressing LINC01764 in HCT116 cells, as indicated, GAPDH was used as the loading control. (E and F) IC_50_ of 5‐FU was significantly decreased when LINC01764 was downregulated while increased when LINC01764 was upregulated in HCT116 and RKO cells. *p < 0.05, **p < 0.01, ***p < 0.001.

To evaluate the biological role of LINC01764 in CRC cells, small interfering RNAs (siRNAs) and LINC01764‐overexpressing lentiviruses were designed to downregulate and upregulate LINC01764, respectively. LINC01764 knockdown efficiently inhibited the proliferation and colony‐formation abilities of HCT116 and RKO cells, whereas LINC01764 upregulation enhanced cell growth (Figures 2A,B and S1A, B). Additionally, LINC01764 downregulation remarkably reduced the migration ability of CRC cells, while its upregulation led to the opposite effects (Figures 2C and FS1C). EMT‐associated and stemness‐associated markers were tested by Western blot. In accordance with the cell migration‐promoting functions of LINC01764, its knockdown markedly reduced the expression of N‐cadherin, Snail, Slug, β‐Catenin, CD133, CD44, and Oct4 while elevating that of E‐cadherin. Conversely, upregulation of LINC01764 exerted the opposite effects (Figures 2D and S1D). Collectively, these results indicate that LINC01764 acts as an oncogene in CRC cells.

As increased LINC01764 expression is related to a poor response to FOLFOX/XELOX treatment and poor prognosis in CRC patients, we assumed that LINC01764 may be related to sensitivity to FOLFOX/XELOX therapy. The FOLFOX regimen comprises folinic acid, fluorouracil, and oxaliplatin, while the XELOX regimen consists of capecitabine and oxaliplatin; capecitabine is a derivative of fluorouracil. Therefore, LINC01764 may be associated with 5‐FU and oxaliplatin sensitivity. Upon determining the half‐maximal inhibitory concentration (IC_50_) of 5‐FU and oxaliplatin, we found that the IC_50_ of 5‐FU was significantly reduced by the knockdown and increased by the overexpression of LINC01764 in HCT116 and RKO cells (Figures 2E,F and S1E,F), while that of oxaliplatin showed no significant changes (data not shown). In addition, we measured the IC_50_ of other chemotherapeutic and targeted drugs, and the results showed that LINC01764 did not affect the drug sensitivity of CRC cells to CPT‐11, cisplatin, and regorafenib (Figure S2). These results suggest that LINC01764 weakens the sensitivity of CRC cells to 5‐FU and may promote 5‐FU resistance.

### 2.3 LINC01764 serves as a promising predictor of chemotherapy response in CRC

**FIGURE 3:**
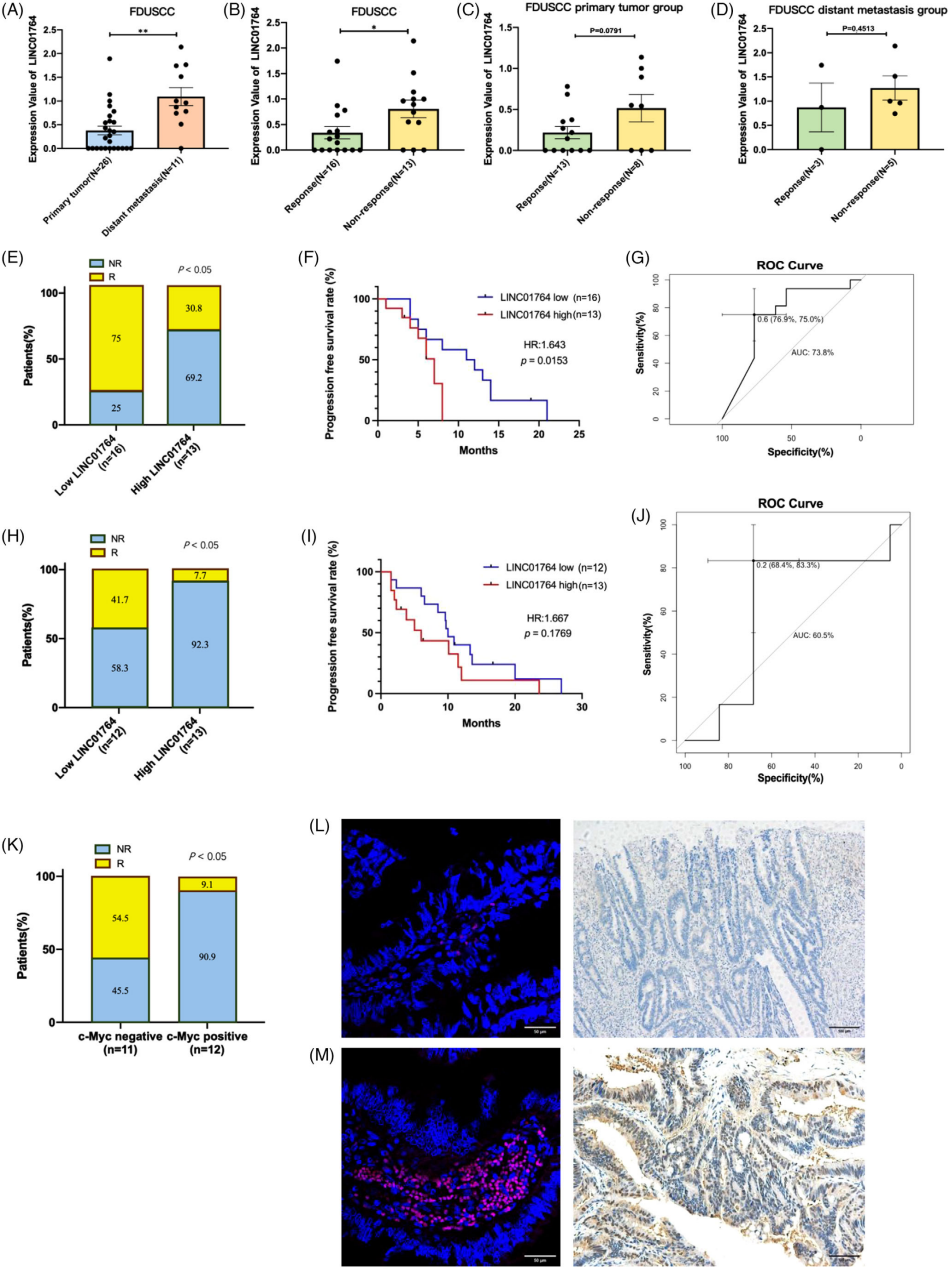
Validation of potential clinical values of LINC01764. (A) Expression of LINC01764 were significantly higher in distant metastasis sample group. (B) Expression of LINC01764 were significantly higher in nonresponse to FOLFOX/XELOX group. (C and D) Analysis of expression difference between different treatment efficacy group in primary tumor samples and distant metastasis samples separately. (E and F) qRT‐PCR analysis was performed and the overall response rate and progression free survival were analyzed between high and low LINC01764 group. (G) The AUROC of LINC01764 for the prediction of chemotherapy response in qRT‐PCR cohort. (H and I) FISH analysis was performed and the overall response rate and progression free survival were analyzed between high and low LINC01764 group. (J) The AUROC of LINC01764 for the prediction of chemotherapy response in FISH cohort. (K) IHC assay was performed and the overall response rate were analyzed between c‐Myc negative and c‐Myc positive group. (L and M) Representative FISH and IHC images. All data are shown as the mean ± SEM values. *p < 0.05, **p < 0.01.

In the 37 biopsy specimens collected, LINC01764 expression was significantly higher in the metastatic samples and FOLFOX/XELOX therapy nonresponse groups than in the primary tumor samples and response groups, demonstrating a correlation among LINC01764, distant metastasis, and poor chemotherapy efficacy (Figure 3A–D).

To clinically verify the utility of LINC01764 in predicting the efficacy of 5‐FU chemotherapy, two cohorts of CRC patients treated with FOLFOX/XELOX chemotherapy were enrolled for validation. One cohort was utilized to detect LINC01764 expression levels in frozen fresh CRC tissue using quantitative reverse transcription‐PCR (qRT‐PCR) and the other to test LINC01764 and c‐Myc protein expression levels in paraffin sections of cancer tissue using FISH and immunohistochemistry, respectively. In the qRT‐PCR‐tested group, with 16 cases of low expression and 13 cases of high expression, the overall response rate (ORR) to 5‐FU chemotherapy in the low‐LINC01764‐expression group was significantly higher than that in the high‐LINC01764‐expression group (75 vs. 30.8%) (Figure 3E). Kaplan–Meier analysis revealed that the LINC01764‐low group had a significantly longer progression‐free survival (*p *= 0.0153) than the LINC01764‐high group (Figure 3F). The predictive power of cancerous LINC01764 was also estimated using the area under the curve (AUC) of the receiver operating characteristic curve, with the AUC value of LINC01764, as tested using PCR, reaching 0.738 (Figure 3G). In the FISH‐tested group with 12 low‐ and 13 high‐expression cases, the ORR to 5‐FU chemotherapy was 41.7%, whereas the high‐expression group demonstrated a significant decrease to 7.7% (Figure 3H). Kaplan–Meier analysis showed that the low LINC01764‐expression group had a longer PFS than the LINC01764‐high group, although the difference was not statistically significant (*p *= 0.1769) (Figure 3I). The AUC of LINC01764, as tested using FISH to predict chemotherapeutic efficacy, was 0.605 (Figure 3J). LINC01764 expression levels (as detected by FISH and qRT‐PCR) were not correlated with clinicopathological parameters (age, sex, gene mutation, differentiation, and tumor location) (Tables S1 and S2). Meanwhile, the ORR was higher in the c‐Myc‐negative group (*N* = 11) than in the c‐Myc‐positive group (*N* = 12) (54.5 vs. 9.1%) (Figure 3K), indicating that c‐Myc was associated with worse responses to chemotherapy. Representative LINC01764 expression images from FISH (the left upper and left lower panels show low and high expression, respectively) and c‐Myc expression images from immunohistochemistry (the right upper and right lower panels show negative and positive expression, respectively) are presented (Figure 3L,M). Collectively, these data suggest that LINC01764 is a promising predictor of the response to chemotherapy in patients with CRC.

### 2.4 LINC01764 performs its oncogenic functions by promoting glucose metabolism and glutamine metabolism

To further explore the functions and underlying mechanisms of LINC01764, we conducted RNA‐seq on cells with stable LINC01764 overexpression and their negative controls. Results revealed that LINC01764 upregulation correlated with the glycolytic pathway (Figure S3A–C). To explore whether LINC01764 functions in glucose metabolism, we performed targeted metabolomic profiling of energy metabolites. Results revealed that upregulation of LINC01764 increased the levels of GAP/DHAP and lactate while reducing those of glucose (Figure S3D). Additionally, the levels of citric acid, fumaric acid, glutamine, and glutamic acid increased (Figure S3E,F). The pathway‐associated metabolite set enrichment analysis indicated that LINC01764 upregulation may activate the Warburg effect, glycolysis, and glutamine metabolism (Figure S3G). Collectively, the results indicated that upregulation of LINC01764 promoted glucose and glutamine metabolism, both hallmarks of cancer metabolic reprogramming.^19^ Subsequently, we focused on glucose metabolism, a crucial aspect of metabolic reprogramming that has gained prominence in research on tumor malignancy, phenotypes, and chemoresistance regulation.^20^, ^21^, ^22^, ^23^, ^24^, ^25^, ^26^ We assessed glucose metabolism levels in HCT116 and RKO cells and found that upregulation of LINC01764 efficiently enhanced glucose uptake, lactate production, and ATP levels, while LINC01764 knockdown yielded opposite results (Figure S3H,I). Western blot further demonstrated that downregulation of LINC01764 remarkably inhibited the expression of HK2, GLUT1, GLUT3, LDHA, and LDHB, whereas its upregulation increased their expression levels (Figure S3J,K). In conclusion, LINC01764 promoted glucose metabolism in CRC cells.

**FIGURE 4:**
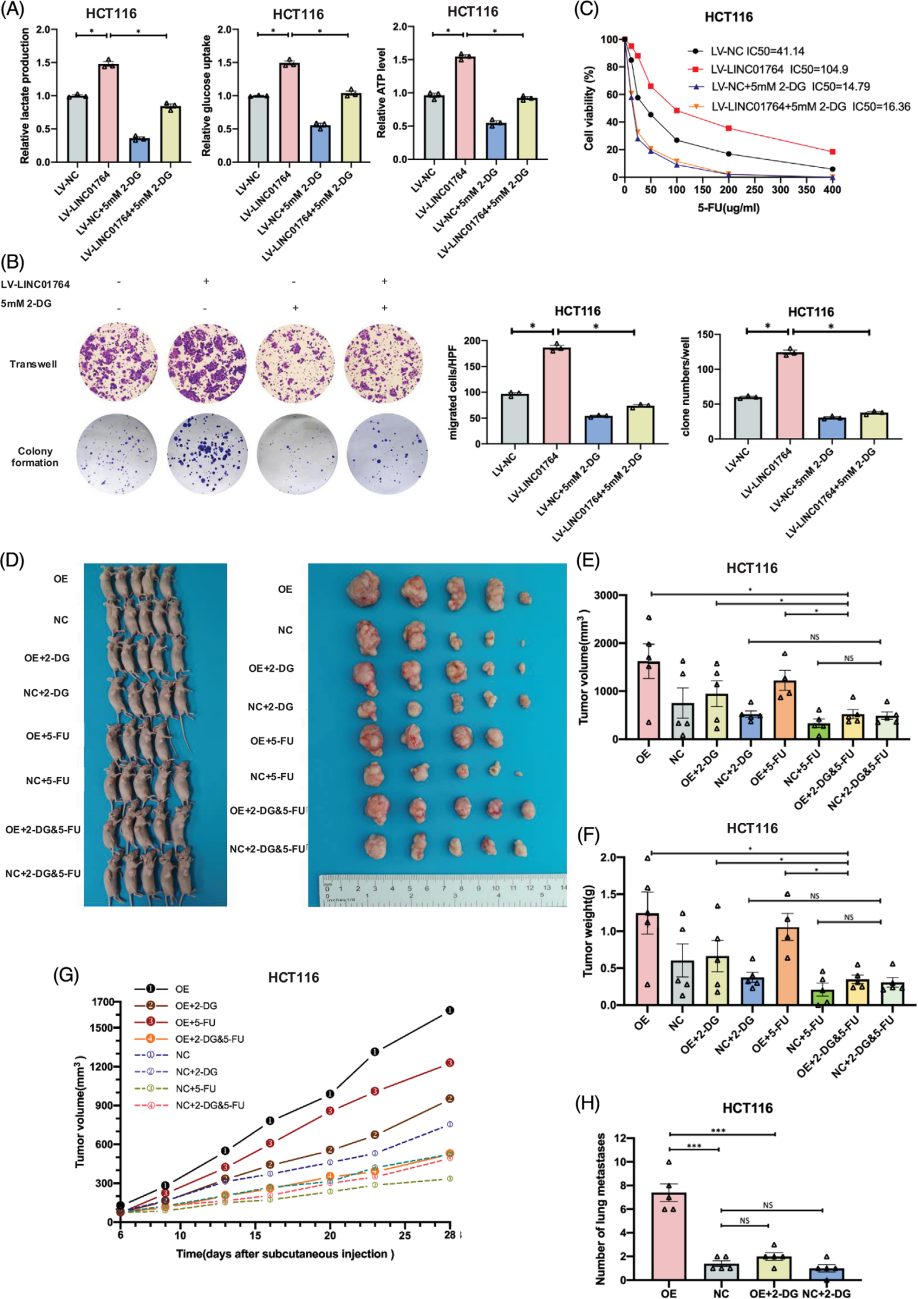
LINC01764 performs its oncogenic functions by promoting glucose metabolism. (A) Measurements of glucose uptake, lactate production and ATP level in HCT116 after LINC01764 overexpressing and 2‐DG treatment. (B and C) Cell proliferation, migration and 5‐FU sensitivity of LINC01764 overexpression cells after 2‐DG treatment were assessed by colony formation (B), Transwell assays (B), and IC_50_ determination (C). 2‐DG treatment eliminated the promoted proliferation, migration, and 5‐FU resistance caused by upregulation of LINC01764 (B and C). Stably transfected HCT116 cells were inoculated into BALB/c nude mice to establish subcutaneous xenograft tumors (*n* = 5 mice/group). Representative images of tumor‐bearing mice (D). Tumor volumes (E), weights (F), and speed of growth (G) of NC, OE, NC+2‐DG, OE+2‐DG, NC+5‐FU, OE+5‐FU, NC+2‐DG and 5‐FU, OE+2‐DG and 5‐FU groups. (H) Stably transfected HCT116 cells were injected into BALB/c nude mice tail vein to establish lung metastasis (*n* = 5 mice/group). Numbers of lung metastases of NC, OE, NC+2‐DG, and OE+2‐DG groups. All data are shown as the mean ± SEM values. *p < 0.05, ***p < 0.001, NS = no significance.

Addition of the glycolysis inhibitor 2‐DG eliminated the increased glucose metabolism induced by LINC01764 overexpression (Figures 4A and S4A). Moreover, 2‐DG treatment eliminated the enhanced proliferation, migration, and 5‐FU resistance caused by LINC01764 upregulation (Figures 4B,C and S4B,C). In a nude mouse subcutaneous xenograft model, upregulation of LINC01764 significantly increased tumor volumes, weights, and growth speed; while treatment with 2‐DG eliminated these effects. However, 5‐FU treatment alone demonstrated no significant efficacy. Notably, the combination of 2‐DG and 5‐FU demonstrated better efficacy than that of either drug alone on LINC01764 overexpressing tumors (Figure 4D–G). The construction of a nude mouse lung metastasis model via tail vein injection revealed that LINC01764 upregulation significantly increased the number of lung metastases, whereas 2‐DG treatment eliminated this effect (Figures 4H and S5). In summary, LINC01764 promotes CRC cell proliferation, metastasis, and 5‐FU resistance, partly by promoting glucose metabolism in vitro and in vivo.

Our preliminary metabolomic analysis also revealed that LINC01764 overexpression increased the intracellular glutamine levels. Hence, we speculated that the LINC01764‐mediated progression and 5‐FU resistance of CRC cells is related to glutamine metabolism. In vitro experiments showed that treatment with the glutamine inhibitor CB839 reversed or partially reversed the promotion of LINC01764 overexpression on the proliferation, migration and 5‐FU resistance of CRC cells (Figure S6A–C). In addition, glutamine levels in the medium and cells were measured 48 h after replacing the fresh medium. The results indicated that cells overexpressing LINC01764 had lower medium glutamine levels and higher intracellular glutamine levels than those in the control group (Figure S6D). Collectively, these data indicate that LINC01764 promotes the proliferation, migration, and 5‐FU resistance of CRC cells via regulating glutamine metabolism.

### 2.5 Biological functions of LINC01764 are mediated through its regulation of c‐Myc protein expression and specifically binding to hnRNPK

**FIGURE 5:**
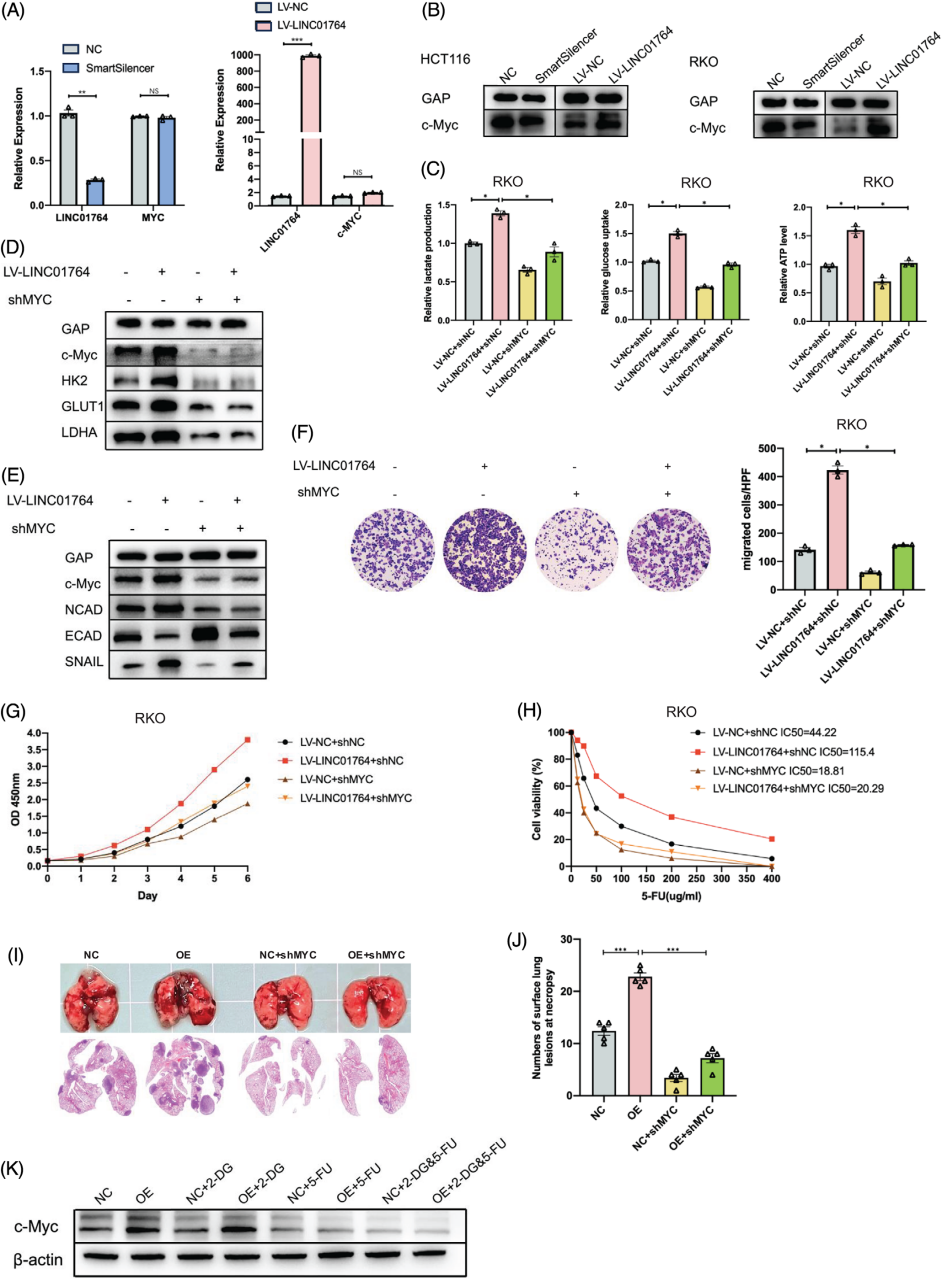
Biological functions of LINC01764 were mediated by its regulation of c‐Myc protein expression level. (A) The expression of c‐MYC mRNA was analyzed by qRT‐PCR after LINC01764 knocking down and overexpressing. (B) The expression of c‐Myc protein was analyzed by Western blot after LINC01764 knocking down and overexpressing. (C) Measurements of glucose uptake, lactate production, and ATP level in RKO cells after cotransfection of LV‐LINC01764 and shMYC. (D and E) Western blot analysis was performed to assess the protein levels of c‐Myc, HK2, GLUT1, LDHA, N‐cadherin, E‐cadherin, and Snail after cotransfection of LV‐LINC01764 and shMYC in RKO cells, as indicated, GAPDH was used as the loading control. (F–H) Cell migration, proliferation and 5‐FU sensitivity of RKO cells after cotransfection of LV‐LINC01764 and shMYC were assessed by Transwell assays (F), CCK‐8 (G), and IC_50_ determination (H). Downregulation of c‐MYC eliminated the promoted proliferation, migration and 5‐FU resistance caused by upregulation of LINC01764. (I and J) Stably transfected HCT116 cells were injected into BALB/c nude mice via tail vein to establish lung metastasis models (*n* = 5 mice/group). Representative images and HE staining of lung metastasis(L). Numbers of surface lung lesions at necropsy(M). (K) Western blot analysis was performed to assess the protein levels of c‐Myc in tumors (obtained from nude mouse subcutaneous xenograft model, which is shown in Figure 4D) of NC, OE, NC+2‐DG, OE+2‐DG, NC+5‐FU, OE+5‐FU, NC+2‐DG and 5‐FU, OE+2‐DG and 5‐FU groups. All data are shown as the mean ± SEM values. *p < 0.05, **p < 0.01, ***p < 0.001.

Gene set expression analysis (GSEA) of datasets from FDUSCC, TCGA, and cell RNA‐seq demonstrated that a high LINC01764 expression correlates with activation of the MYC signaling pathway (Figure S7A–C). Western blot revealed that LINC01764 overexpression increased c‐Myc protein levels, whereas its knockdown suppressed its expression, with no significant changes observed in c‐MYC mRNA expression (Figure 5A,B). These results indicate that LINC01764 promotes c‐Myc protein expression. To evaluate the effect of c‐Myc protein expression regulation on the biological functions of LINC01764, we designed a small hairpin RNA (shRNA) targeting c‐MYC. After cotransfection of LV‐LINC01764 and shMYC, Glucose metabolism measurements revealed that the enhancement of glucose uptake, lactate production, and ATP levels induced by LINC01764 upregulation was inhibited by c‐MYC knockdown (Figure 5C). Western blot analysis revealed a significant reduction in c‐Myc protein expression, accompanied by inhibition of the LINC01764‐induced upregulation of HK2, GLUT1, LDHA, N‐cadherin, E‐cadherin, and Snail (Figure 5D,E). Moreover, c‐MYC knockdown eliminated the promotion of proliferation, migration, and 5‐FU resistance caused by LINC01764 upregulation in vitro (Figure 5F–H). A nude mouse lung metastasis model constructed by tail vein injection showed that c‐MYC knockdown could completely reverse the metastasis‐promoting effect of LINC01764 (Figure 5I,J). Furthermore, in nude mouse subcutaneous xenograft model, which is shown in Figure 4D, overexpression of LINC01764 upregulated c‐Myc protein, whereas 5‐FU treatment downregulated c‐Myc protein expression, and 2‐DG treatment did not affect c‐Myc expression (Figure 5K).

As LINC01764 is mainly localized in the nuclei, this action likely involves interactions with RNA‐binding protein (RBP). Although c‐Myc itself is not an RBP, RNA pull‐down assays were performed to explore its potential mechanism of action. We found that heterogeneous nuclear ribonucleoprotein K (hnRNPK), an oncogenic protein whose downstream genes include c‐MYC,^27^ specifically bound to the LINC01764 sense strand. This interaction was further validated through radioimmunoprecipitation (RIP) assays (Figure S8A,B). To explore whether LINC01764 regulates c‐Myc protein expression by binding to hnRNPK, we designed an shRNA targeting hnRNPK. Cotransfection of LV‐LINC01764 and sh‐hnNRNPK resulted in the inhibition of c‐Myc protein upregulation induced by LINC01764 overexpression, whereas c‐MYC mRNA expression was not significantly altered (Figure S8C–E). Moreover, the glucose metabolism, proliferation, migration, and 5‐FU resistance enhanced by LINC01764 upregulation were inhibited by hnRNPK knockdown (Figure S8F–I).

### 2.6 LINC01764 promotes the IRES‐dependent translation of c‐MYC by binding to hnRNPK

**FIGURE 6:**
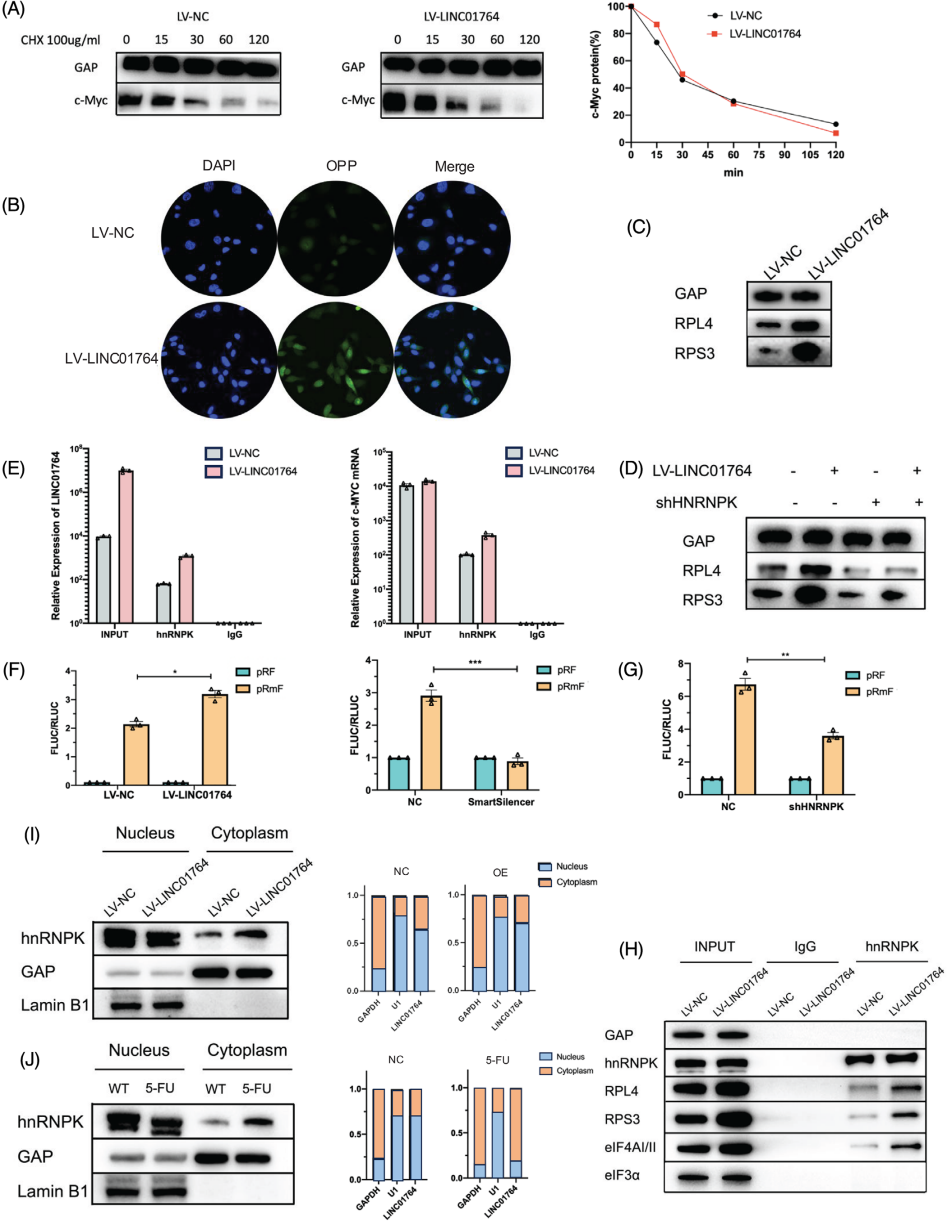
LINC01764 promoted IRES‐dependent translation of c‐MYC by binding to hnRNPK. (A) Western blot analysis was performed to assess the half‐life of c‐Myc after LINC01764 overexpressing. (B) OPP protein synthesis assay was conducted to detect and visualize the protein synthesis in CRC cells. Green fluorescence indicates newly synthesized proteins. Nuclei were stained with DAPI (blue). (C) Western blot analysis was performed to assess the protein levels of RPL4 and RPS3 after LINC01764 overexpressing. (D) Western blot analysis was performed to assess the protein levels of RPL4 and RPS3 after cotransfection of LV‐LINC01764 and shHNRNPK. (E) RIP assays were performed to assess the binding of hnRNPK to LINC01764 and c‐MYC after LINC01764 overexpression. (F) Dual‐luciferase reporter assay was performed to assess the activity of c‐MYC IRES after LINC01764 overexpression and knocking down. (G) Dual‐luciferase reporter assay was performed to assess the activity of c‐MYC IRES after HNRNPK knocking down. (H) IP assays were performed to assess the binding of hnRNPK to ribosomal protein and translation initiation factor after LINC01764 overexpression. (I) qRT‐PCR and Western blot analysis were performed to evaluate the location of LINC01764 and hnRNPK after LINC01764 overexpression. (J) qRT‐PCR and Western blot analysis were performed to evaluate the location of LINC01764 and hnRNPK after 5‐FU treatment. All data are shown as the mean ± SEM values. *p < 0.05.

To explore the specific mechanism by which LINC01764 regulates c‐Myc expression, we examined the half‐life of the c‐Myc protein and found that upregulation of LINC01764 had no significant effect on the half‐life of the c‐Myc protein (Figure 6A), suggesting that LINC01764 regulates c‐Myc expression at the translational level, rather than the post‐translational level. Therefore, we measured protein synthesis levels, which revealed that LINC01764 upregulation markedly enhanced protein synthesis (Figure 6B). In LINC01764‐overexpressing cells, the expression of ribosomal proteins ribosomal protein L4 (RPL4) and ribosomal protein S3 (RPS3) was enhanced, which was attenuated upon the simultaneous downregulation of hnRNPK (Figure 6C,D). RIP assays indicated that hnRNPK specifically binds to c‐MYC mRNA. Moreover, LINC01764 upregulation increased its degree of binding to hnRNPK, and the binding of hnRNPK to c‐MYC mRNA was markedly increased (Figure 6E). Dual‐luciferase reporter assays indicated that the upregulation of LINC01764 significantly stimulated internal ribosome entry site (IRES) activity, which was inhibited by the downregulation of LINC01764 and hnRNPK (Figure 6F,G). Immunoprecipitation (IP) assays indicated that LINC01764 upregulation promoted the binding of hnRNPK to ribosomal proteins (RPS3 and RPL4) and translation initiation factors (eIF4AI/II) (Figure 6H). Moreover, LINC01764 upregulation promoted hnRNPK nuclear egress (Figure 6I), and 5‐FU treatment promoted both hnRNPK and LINC01764 nuclear egress (Figure 6J). These results indicate that LINC01764 binds to hnRNPK in the nucleus, facilitating its transport to the cytoplasm where it binds to c‐MYC IRES and promotes its translation.

### 2.7 LINC01764 regulates uridine phosphorylase 1 mRNA expression by promoting the c‐MYC, glucose, and glutamine metabolism pathways

**FIGURE 7:**
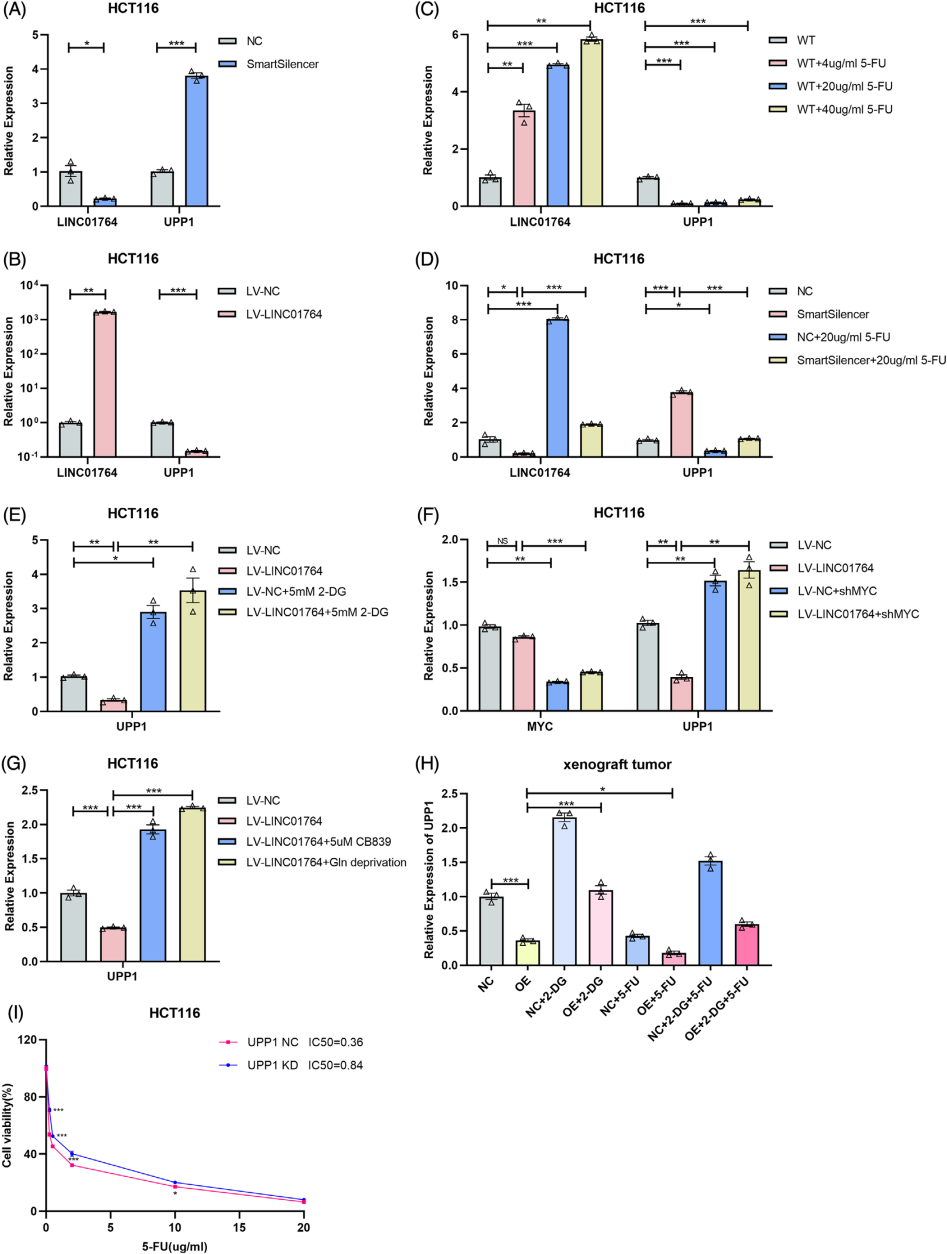
LINC01764 regulates *UPP1* mRNA expression by promoting c‐MYC and glucose metabolism pathways. (A) Knockdown of LINC01764 significantly enhanced *UPP1* mRNA expression. (B) Overexpression of LINC01764 significantly decreased *UPP1* mRNA expression. (C and D) 5‐FU treatment upregulated LINC01764 expression and downregulated *UPP1* expression. HCT116 cells were treated with 0, 4, 20, 40 µg/mL 5‐FU for 24 h (C) and LINC01764‐knockdown cells treated with 20 µg/mL 5‐FU for 24 h before harvesting (D). (E) Inhibition of glucose metabolism promoted *UPP1* mRNA expression. HCT116 cells overexpressing LINC01764 and control cells were treated with or without 5 mM 2‐DG for 24 h before harvesting. (F) Knockdown of c‐MYC elevated *UPP1* expression. (G) Inhibition of glutamine metabolism enhanced *UPP1* expression. HCT116 cells overexpressing LINC01764 cells were treated with 5uM CB839 or glutamine deprivation for 48 h before harvesting. (H) *UPP1* mRNA in tumors (obtained from nude mouse subcutaneous xenograft model, which is shown in Figure 4E) of NC, OE, NC+2‐DG, OE+2‐DG, NC+5‐FU, OE+5‐FU, NC+2‐DG and 5‐FU, OE+2‐DG and 5‐FU groups. (I) IC_50_ of 5‐FU was significantly increased when *UPP1* was downregulated in HCT116 cells. LINC01764 and *UPP1* mRNA expression were detected by qRT‐PCR. All data are shown as the mean ± SEM values. *p < 0.05, **p < 0.01, ***p < 0.001, NS = no significance.

To determine the mechanism by which LINC01764 promotes 5‐FU resistance in CRC cells, we performed RNA sequencing of HCT116 cells overexpressing LINC01764 and control cells. Notably, uridine phosphorylase 1 (*UPP1*) was significantly downregulated in LINC01764‐overexpressing cells. Additionally, we focused on *UPP1* for the following two reasons: (i) *UPP1* facilitates the conversion of 5‐FU to FUMP and FdUTP and (ii) *UPP1*‐knockout mouse embryonic stem cells are 10‐fold more resistant to 5‐FU, whereas *UPP1* overexpression enhances its cytotoxicity. qRT‐PCR analysis showed that LINC01764 negatively regulates *UPP1* mRNA expression (Figure 7A,B). Moreover, 5‐FU treatment upregulated LINC01764 and downregulated *UPP1*, with LINC01764 levels being positively correlated with 5‐FU concentration (Figure 7C), and 5‐FU treatment reversed the upregulation of *UPP1* by LINC01764 downregulation (Figure 7D). Additionally, 2‐DG treatment and c‐MYC knockdown reversed the downregulation of *UPP1* mRNA by LINC01764 overexpression (Figure 7E,F). Both CB839 (a glutamine inhibitor) treatment and glutamine deprivation induced higher expression of *UPP1* in LINC01764‐overexpressing cells than in the controls (Figure 7G). In nude mouse subcutaneous xenograft model, which was shown in Figure 4E, both overexpression of LINC01764 and 5‐FU treatment downregulated *UPP1* mRNA, whereas 2‐DG treatment upregulated *UPP1* expression (Figure 7H). Additionally, IC_50_ assay indicated that knockdown of *UPP1* significantly increased the IC_50_ of 5‐FU in HCT116 cells (Figure 7I). However, knockdown of *UPP1* did not reverse the inhibitory effect on cell proliferation and migration caused by LINC01764 downregulation (Figure S4A–C), indicating that these functions of LINC01764 was not mediated by *UPP1*. Collectively, these data suggested that LINC01764 induced 5‐FU chemoresistance by promoting the c‐MYC, glucose metabolism, and glutamine metabolism pathways to downregulate *UPP1*, subsequently reducing the conversion of 5‐FU to the active form.

## 3 DISCUSSION

**FIGURE 8:**
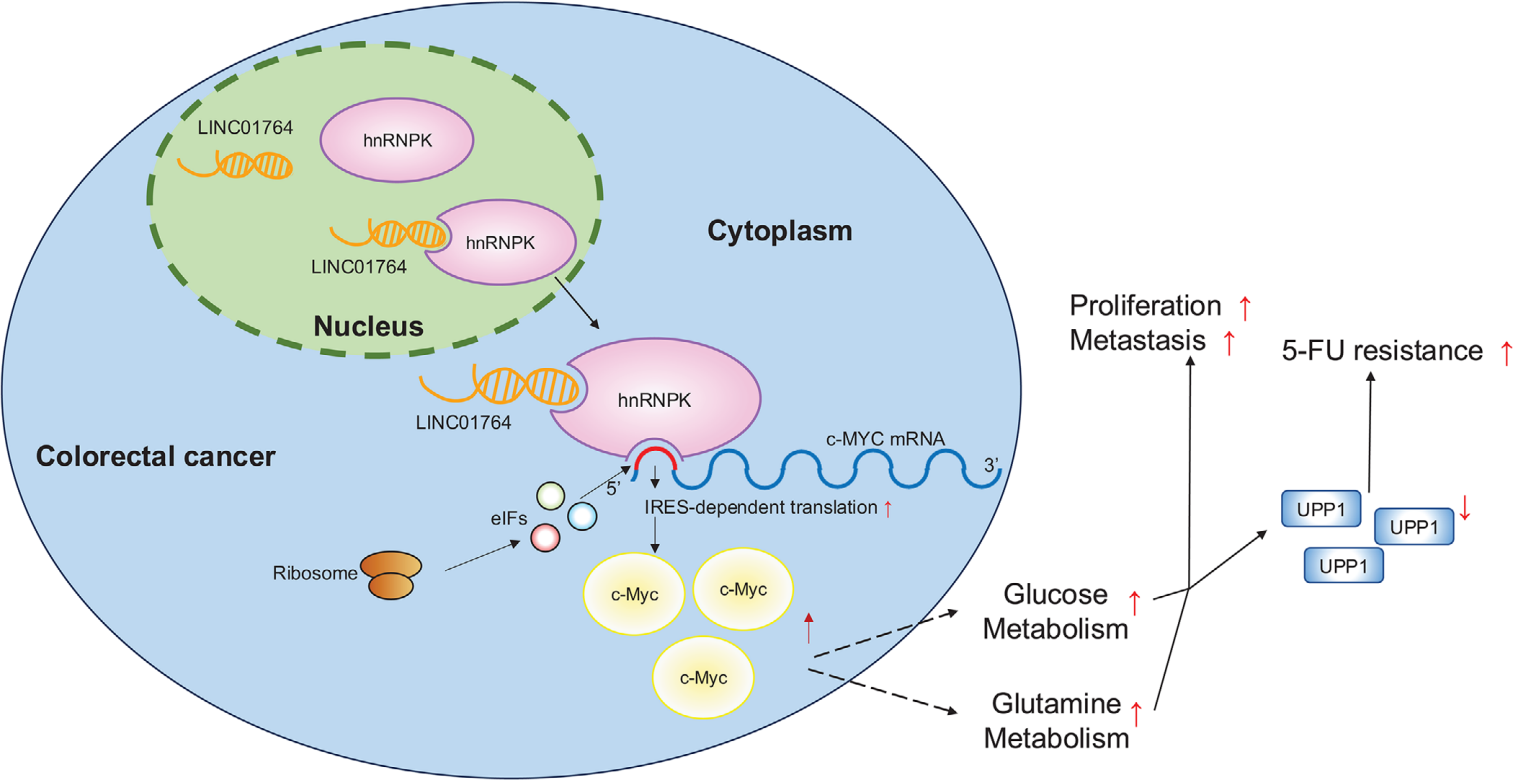
Working model of LINC01764 in CRC.

Researches have confirmed that the abnormal expression of numerous lncRNAs is closely related to various pathophysiological processes in CRC, such as metastasis and chemoresistance. Despite this understanding, only a few lncRNAs have transitioned into clinical applications such as cancer screening, prognosis, or chemotherapy response prediction. To the best of our knowledge, this study is the first to report the potential clinical value of the LINC01764/hnRNPK/c‐MYC axis in CRC progression and 5‐FU resistance. LINC01764 recruits hnRNPK to the c‐MYC IRES, which subsequently recruits translation initiation factors and interacts with ribosomal proteins to promote c‐MYC translation. Upregulation of c‐MYC promotes glucose and glutamine metabolism; thus, *UPP1* is downregulated, and 5‐FU resistance is enhanced (Figure 8).

Through bioinformatics analysis of RNA‐seq data from the TCGA‐COAD dataset, we identified lncRNAs significantly upregulated in metastatic samples and chemotherapy‐resistant patients and confirmed that LINC01764 was overexpressed in CRC tissues and cell lines and correlated with distant metastasis and shorter disease‐free survival. These results indicated the potential oncogenic characteristics of LINC01764. Functional experiments demonstrated that LINC01764 acts as an oncogene to promote the proliferation, migration, and 5‐FU resistance of CRC cells in vitro.

Our GSEA results prompted further investigation into the relationship between LINC01764 and glycolysis. The enhancement of glucose metabolism in malignant tumors is beneficial for energy supply, biosynthesis, signal transduction, and many other functions crucial for tumor cell survival, proliferation, migration, and invasion.^19^, ^20^, ^21^ In addition, enhanced glucose metabolism contributes to chemoresistance.^22^, ^23^, ^24^ Metabolomics and measurements of cell glucose uptake, lactate production, and ATP levels confirmed that LINC01764 promotes glucose metabolism. Furthermore, 2‐DG treatment demonstrated that LINC01764 promotes CRC cell proliferation, migration, and 5‐FU resistance by promoting glucose metabolism both in vivo and in vitro. Additionally, different responses to 2‐DG treatment were observed between tumors overexpressing LINC01764 and the negative controls, suggesting that upregulation of LINC01764 may enhance sensitivity to 2‐DG treatment in vivo because of elevated glucose metabolism levels. Moreover, in LINC01764 overexpressing tumors, 5‐FU combined with 2‐DG showed better antitumor effects, which providing a basis for future clinical research using 5‐FU combined with glycolysis inhibitors to treat tumors with high glycolysis level and LINC01764 as a biomarker of sensitivity to glycolysis inhibitor treatment.

The regulation of c‐MYC could be achieved through multiple levels. A previous study showed that UTP14a could stabilize c‐MYC and protect c‐MYC from degradation through the ubiquitination–proteasome pathway.^28^ We observed an association between LINC01764 and the MYC signaling pathway, which showed that LINC01764 positively regulated c‐MYC protein expression rather than c‐MYC mRNA expression. c‐MYC, a classical and well‐characterized proto‐oncogene, triggers metabolic reprogramming in cancer cells. Various glycolytic enzymes, including LDHA, HK2,^29^ and GLUT1,^30^ are directly regulated by c‐MYC, which is consistent with our finding that LINC01764 modulates the expression of glycolytic enzymes by regulating c‐MYC. Rescue experiments confirmed that the biological functions of LINC01764 were mediated by the regulation of c‐MYC protein expression. LncRNAs localized in the nuclei play regulatory roles mainly by interacting with RBP. RNA pull‐down and RIP assays confirmed that LINC01764 specifically binds to hnRNPK, which participates in various physiological and pathophysiological processes, including spermatogenesis, neural system development, red blood cell differentiation, organogenesis, and carcinogenesis.^31^, ^32^, ^33^ Recently, the interactions between hnRNPK and lncRNAs have been suggested to be associated with tumorigenesis.^34^, ^35^, ^36^ In this study, we demonstrated that the specific binding of LINC01764 to hnRNPK upregulated c‐MYC protein expression.

Previous studies have demonstrated that hnRNPK could bind to and activate the IRES in the 5ʹUTR region of *c‐MYC* mRNA, promoting the IRES‐dependent translation of c‐MYC.^27^ IRES is a unique segment that directly recruits small ribosomal subunits and drives protein synthesis; furthermore, many IRES‐containing mRNAs are involved in cancer cell growth, apoptosis, and angiogenesis.^37^, ^38^, ^39^, ^40^, ^41^ The existence of an IRES element in the 5ʹUTR region of c‐MYC mRNA has already been confirmed,^42^ and IRES‐dependent translation of c‐MYC is related to breast cancer, multiple myeloma, and other cancers.^43^, ^44^ Using RIP and dual‐luciferase reporter assays, we demonstrated that the specific binding of LINC01764 to hnRNPK enhanced the binding of hnRNPK to c‐MYC IRES and stimulated IRES activity, thus promoting IRES‐dependent c‐MYC translation.

Apart from glucose metabolic reprogramming, cancer cells also exhibit a substantially increased demand for glutamine, termed as “glutamine addiction.”^45^ Glutamine, a major amino acid, is metabolically transformed to α‐ketoglutarate; then, α‐ketoglutarate enters the tricarboxylic acid cycle to generate ATP, serving as a fuel for the rapid proliferation of cancer cells.^46^ Extensive studies have confirmed that glutamine facilitates CRC cell proliferation, migration, and metastasis and participates in 5‐FU chemoresistance.^47^ Moreover, the STAT3–MYC axis regulates the neutral amino acid transporter SLC1A5, controlling the influx of glutamine into acute myeloid leukemia cells, maintaining their energy metabolism and survival.^48^ Notably, glutamine reportedly stabilizes c‐MYC via α‐ketoglutarate to prevent c‐MYC degradation through the ubiquitination–proteosomal pathway, thus weakening the sensitivity of pancreatic cancer cells to paclitaxel.^49^ These studies indicate that c‐MYC significantly participates in the glutamine metabolism, supporting our finding that LINC01764 upregulates c‐MYC and promotes cell proliferation, migration, and attenuates 5‐FU resistance in CRC cells by regulating glutamine metabolism.

Previous studies revealed that glycolysis could inhibit oxidative phosphorylation and decrease ROS production.^45^ Conversely, CB‐839 treatment could inhibit glutamine metabolism and increase ROS levels.^50^ Therefore, both glycolysis and glutamine metabolism cause a reduction in ROS levels, and ROS can activate Nrf2 and increase *UPP1* levels, thereby facilitating the conversion of 5‐FU into its active compound.^45^ Our experiments revealed that LINC01764 overexpression leads to lower *UPP1* levels. Based on these facts, we predicted that LINC01764 promotes glucose and glutamine metabolism, thus reducing ROS levels and, in turn, decreasing *UPP1* expression, the levels of 5‐FU active compounds, and finally increasing chemoresistance. These results may explain the mechanism underlying the role of LINC01764 in 5‐FU resistance.

Our study has some limitations. First, as the number of tissue sample is limited, the prognostic and chemotherapy efficacy predictive value of LINC01764 requires validation with a larger sample size. Second, it remains to be clarified whether other molecules in this pathway have prognostic and therapeutic predictive value. Third, the downstream mechanisms of c‐MYC in this pathway require further in‐depth investigation in the future.

In conclusion, LINC01764 specifically binds to hnRNPK to regulate the IRES‐dependent translation of c‐MYC. High LINC01764 expression correlates with distant metastasis, a poor response to FOLFOX/XELOX therapy, and unfavorable prognoses in patients with colon cancer. Moreover, LINC01764 enhances glycolysis and glutamine metabolism to promote CRC cell proliferation, metastasis, and 5‐FU resistance both in vitro and in vivo. Mechanistically, LINC01764 induces 5‐FU chemoresistance by upregulating the c‐MYC and glucose metabolism pathways, leading to the downregulation of *UPP1* and subsequent reduction in 5‐FU activation. Therefore, LINC01764 holds promise as a predictor of chemotherapy response in CRC. This research not only enhances our understanding of CRC cells metastasis and 5‐FU chemotherapy resistance but also highlights potential clinical applications.

## 4 MATERIALS AND METHODS

### 4.1 Tissue specimens

Tissue samples, including unpaired primary tumor and distant metastasis samples, were collected from 62 patients with advanced CRC. All patients were treated at the FDUSCC between 2019 and 2021, with 47 receiving FOLFOX/XELOX treatment. Written informed consent was obtained from all the participants. The pathological diagnoses of all the patients were independently confirmed by two pathologists. This study was approved by the Ethics Committee of FDUSCC, and the acquisition and application of tissue specimens strictly adhered to ethical principles.

### 4.2 Cell lines and cell culture

NCM460, DLD‐1, HCT‐8, LoVo, HCT‐15, HT‐29, HCT116, SW480, SW620, SW1116, RKO, and P53R cell lines were procured from the Cell Bank of the Chinese Academy of Sciences. All cells were cultured in Dulbecco's modified Eagle's medium (DMEM) (Basalmedia Technologies Co., Shanghai, China), except HCT116 cells, which were cultured in McCoys 5A medium (Gibco, Waltham, MA, USA) supplemented with 10% fetal bovine serum (FBS). Cells were cultured in a suitable cell incubator at 37°C under 5% CO_2_. Short tandem repeat analysis was employed to authenticate all cell lines used in our experiments within the last 3 years.

### 4.3 RNA extraction and RNA‐seq

Total RNA was extracted from CRC tissues and cells using TRIzol Reagent (Invitrogen, Carlsbad, CA, USA) following the manufacturer's instructions. cDNA libraries were constructed following the instructions for Illumina RNA‐Seq preparation. cDNA fragments were amplified and sequenced at both ends using PCR and Illumina Genome Analyzer IIx.

### 4.4 Quantitative reverse transcription‐PCR

RNA was reverse transcribed using the PrimeScript RT Reagent Kit with gDNA Eraser (Takara, Kusatsu, Japan). qRT‐PCR was performed on a QuantStudio 7 Flex System (Thermo Fisher Scientific, Waltham, MA, USA) using TB Green Premix Ex Taq II (Tli RnaseH Plus; Takara). Each sample was tested thrice, and the experiment was performed in triplicate. Analysis was performed using the 2^−ΔΔCt^ method. *GAPDH* was used as the endogenous control for measuring lncRNA and mRNA expression levels. All primers were purchased from Tsingke Biotech (Shanghai, China).

### 4.5 FISH assay

For FISH assays, cells were plated and grown in confocal dishes until cell coverage reached at least 60%. After washing the dishes twice with PBS, cells were fixed with 4% paraformaldehyde and permeabilized with 0.5% Triton X‐100. A FISH probe mix targeting LINC01764 and U6, that is, 18S (RiboBio, Guangzhou, China), was denatured and incubated with cells at 37°C overnight. The next day, DAPI was used to stain the cell nuclei for 15 min after hybridization. Subsequently, images were obtained using a confocal fluorescence microscope (Leica, Wetzlar, Germany).

### 4.6 Lentivirus infection and transfection

Lentiviruses and scrambled (negative control) LINC01764 for in vivo experiments were acquired from Hanyin Biotechnology Limited Company (Shanghai, China). Stable cell lines were obtained after treatment with 4 µg/mL puromycin for 72 h. SmartSilencer (siRNA) against LINC01764 (si‐LINC01764) and siRNA control were synthesized by RiboBio Co. The shRNAs for c‐MYC and hnRNPK were obtained from Hanyin Biotechnology Ltd. The jetPRIME transfection reagent (Polyplus‐transfection SA, Strasbourg, France) was used to transfect siRNA or shRNA into CRC cells (RKO, HCT‐116) according to the manufacturer's protocol. The transfection efficiency was determined using qRT‐PCR.

### 4.7 Cell migration assays

For the Transwell assays, chambers with 8.0 µm pores (Corning, Corning, NY, USA) were used. We added 600 µL medium containing 20% FBS into each well of the 24‐well plates as a cell nutritional attractant. Then, 200 µL of a serum‐free cell suspension (5 × $10^{4}$ cells) was added into the upper chamber. Following incubation for 24−48 h, cells on the inner side of the chamber were removed using a cotton swab. Next, the migrated cells were fixed with 4% polyformaldehyde and stained with 0.2% crystal violet. Subsequently, images of the migrated cells were captured using an inverted microscope (Olympus, Tokyo, Japan).

### 4.8 Cell proliferation assays

Cell viability was evaluated using the CCK‐8 reagent (Yeasen, Shanghai, China). First, 2 × 10^3^ cells were seeded into each well of 96‐well plates. Next, 100 µL CCK‐8 working solution (CCK‐8: medium = 1:10) was added per well, followed by incubation for 2 h. Finally, cell quantity was analyzed by gauging the absorbance at 450 nm using an absorbance reader (Biotek, Winooski, VT, USA). Colony formation assays were performed as previously described.^51^

### 4.9 IC_50_ determination

Cells were seeded and treated with different concentrations of oxaliplatin and 5‐FU. After incubation for 48 h, 100 µL CCK‐8 working solution was added to each well and incubated for 2 h. The cellular content was quantified by measuring the absorbance at 450 nm using an absorbance reader. The GraphPad Prism software was used to calculate the IC50 of 5‐FU.

### 4.10 Western blot analysis

Proteins were extracted from cells using RIPA buffer augmented with proteinase and phosphate inhibitors. Protein concentration was measured using the BCA reagent (Thermo Fisher Scientific). Target proteins were separated via 10% sodium dodecyl sulfate‐polyacrylamide gel electrophoresis (SDS‐PAGE) and subsequently transferred onto PVDF membranes (Millipore, Burlington, MA, USA). The membranes were blocked for 1 h with 5% skim milk. Next, the membranes were immunoblotted with primary antibodies overnight at 4°C and secondary antibodies for 1 h at room temperature. The targeted proteins were visualized using enhanced chemiluminescence reagent (Millipore) and mageQuant LAS 4000 (GE Healthcare Life Sciences, Chicago, IL USA). GAPDH was used as the loading control.

### 4.11 Metabolomics

Targeted metabolomic profiling of the energy metabolites was performed using a UPLC–MS/MS platform (Metabo‐Profile, Shanghai, China). Sample preparation followed previously published methods with slight adjustments. The experiments were performed in six replicates.

### 4.12 Measurement of glucose uptake, lactate production, ATP levels, and glutamine concentration

Glucose uptake, lactate production, ATP levels, and glutamine metabolism were measured using glucose uptake colorimetric (BioVision, Milpitas, CA, USA), lactate colorimetric (BioVision), firefly luciferase‐based ATP (Beyotime), and glutamine assay kits (BioVision). All experiments were performed according to the manufacturer's instructions.

### 4.13 Animal experiments

All animal experiments were approved by the Institutional Animal Care and Use Committee (IACUC) of FDUSCC. Female BALB/c nude mice (5 weeks old) were obtained. For the tumor growth study, the following eight groups (*N* = 5 mice/group) were established: NC/OE without treatment, with 2‐DG treatment, with 5‐FU treatment, and with 2‐DG and 5‐FU treatment. Mice were inoculated with cells dissolved in 200 µL PBS to generate xenograft tumors and treated as planned until the xenografts grew to visible tumors. Tumor dimensions were measured thrice weekly, and tumor weight was assessed 30 days after cell inoculation. For the tumor metastasis study, the following eight groups (*N* = 5 mice/group) were established: NC/OE without and with 2‐DG treatment, NC/OE without and with shMYC cotransfection. Mice were injected with cells dissolved in 200 µL PBS through tail veins to generate a lung metastasis model and were treated as planned 3 days after injection. The lungs were excised, any visible metastases were counted and recorded, and the lungs were fixed with 4% polyformaldehyde for hematoxylin and eosin staining.

### 4.14 RNA pull‐down assay

Pull‐down assays were performed using a pierce magnetic RNA‐protein pull‐down kit (Thermo Fisher Scientific) following the manufacturer's instructions. RBP complexes were separated using SDS‐PAGE, and protein mass spectrometry was performed to identify proteins with specific binding capabilities.

### 4.15 RNA immunoprecipitation

RIP assays were conducted using the Magna RIP Kit (Millipore) according to the manufacturer's instructions. The immunoprecipitated RNA was purified and analyzed using qRT‐PCR. RIP assays were performed in LINC01764‐overexpressing cells and their corresponding negative controls.

### 4.16 Protein synthesis assay

Protein synthesis assays were performed using the Click‐iT Plus OPP Protein Synthesis Assay Kit (Invitrogen) according to the manufacturer's instructions. Protein synthesis was visualized using a confocal fluorescence microscope (Leica).

### 4.17 c‐MYC IRES activity

The pRF reporter construct was synthesized, and c‐MYC IRES was incorporated into the pRF to produce pRmF. Both pRF and pRmF were obtained from Hanyin Biotechnology, Ltd. Reporter constructs were transfected into lines using FuGENE HD Transfection Reagent (Promega, Madison, WI, USA) to achieve a transfection efficiency of 5−10%. After 18 h, the cells were harvested and luciferase activity was measured using a dual‐luciferase reporter assay system (Promega). All luciferase activities were normalized to the luciferase values obtained for pRF.

### 4.18 Statistical analysis

Statistical analyses were performed using R version 3.6.1, SPSS Statistics 26.0, and GraphPad Prism 8.0. Differences between two groups were analyzed using Student's *t*‐test. Survival curves were constructed using the Kaplan–Meier method, and significance was evaluated using the log‐rank test. Spearman's rank correlation test was used to analyze the correlation between two indicators. A paired‐sample *t*‐test was used to analyze the differences between paired groups. Statistical significance was set at *p* < 0.05 for all tests. Results are presented as the mean ± SEM of values from at least three independent experiments.

## AUTHOR CONTRIBUTIONS

Weijian Guo designed and supervised this study. Ran Duan wrote the main text of the manuscript, conducted most of the in vitro and in vivo experiments, and performed data analyses. Yujia Zhai conducted experiments to explore the mechanism underlying LINC01764‐modulated 5‐FU resistance and performed part of the in vitro and in vivo experiments. Qiushuang Wang conducted some of the experiments, collected and analyzed the clinical data, and contributed to manuscript writing. Liqin Zhao, Yixuan Wang, Nuoya Yu, and Jieyun Zhang participated in the experiments and data analysis. All the authors have read and approved this manuscript.

## CONFLICT OF INTEREST STATEMENT

The authors declare that there are no conflict of interests.

## ETHICS STATEMENT AND CONSENT TO PARTICIPATE

The collection of clinical data and tissue specimens was explained to all patients, and written informed consent was obtained from all participants. Tissue specimens used in this study were approved by the Ethics Committee of the FDUSCC (approval number: 050432‐4‐2108). For animal studies, a special approval (FUSCC‐IACUC‐2022034) from the IACUC of FDUSCC was obtained since the subcutaneous tumor volume of one mouse in the linc01764‐overexpression group exceeded 2000 mm^3^.

## Supporting information



Supporting Information

## Data Availability

Data used in this study are available from the corresponding author upon request.
