# Supplementary material for: LINC01764 promotes colorectal cancer cells proliferation, metastasis, and 5‐fluorouracil resistance by regulating glucose and glutamine metabolism via promoting c‐MYC translation
Source: MedComm (2020). 2024 Nov 11;5(11):e70003. doi: 10.1002/mco2.70003 (PMC11555016; doi:10.1002/mco2.70003)
Supplement: Supplementary file 1 — Supporting Information [file MCO2-5-e70003-s001.pdf]

LINC01764 promotes colorectal cancer cells proliferation, metastasis and 5-Fluorouracil resistance by regulating glucose and glutamine metabolism via promoting c-MYC translation

Ran Duan[1,2,3]*, Yujia Zhai[1,2]*, Qiushuang Wang[1,2]*, Liqin Zhao[4], Yixuan Wang[1,2], Nuoya Yu[1,2], Jieyun Zhang[1,2], Weijian Guo[1,2]#

1 Department of Gastrointestinal Medical Oncology, Fudan University Shanghai Cancer Center, Shanghai, China

2 Department of Oncology, Shanghai Medical College, Fudan University, Shanghai, China

3 Department of Medical Oncology, Fujian Cancer Hospital and Fujian Medical University Cancer Hospital, Fujian Medical University, Fuzhou, China

4 Department of Oncology, Ruijin Hospital, Shanghai Jiao Tong University School of Medicine, Shanghai, China

Running title: LINC01764 promotes 5-Fluorouracil resistance in colorectal cancer.

*These authors have contributed equally to this work

#Correspondence to : Weijian Guo, e-mail: guoweijian1@hotmail.com, Tel: 86-21-64175590, Fax: 86-21-64170366

Authors Note:

Ran Duan: 13301050258@fudan.edu.cn

Yujia Zhai: zz1090054601@163.com

Qiushuang Wang: wqs1027@163.com

Liqin Zhao: zlq12405@rjh.com.cn

Yixuan Wang: wyxmedicine@163.com

Nuoya, Yu: nyyu15@fudan.edu.cn

Jieyun Zhang: zjy000999@163.com

Weijian Guo: guoweijian1@hotmail.com

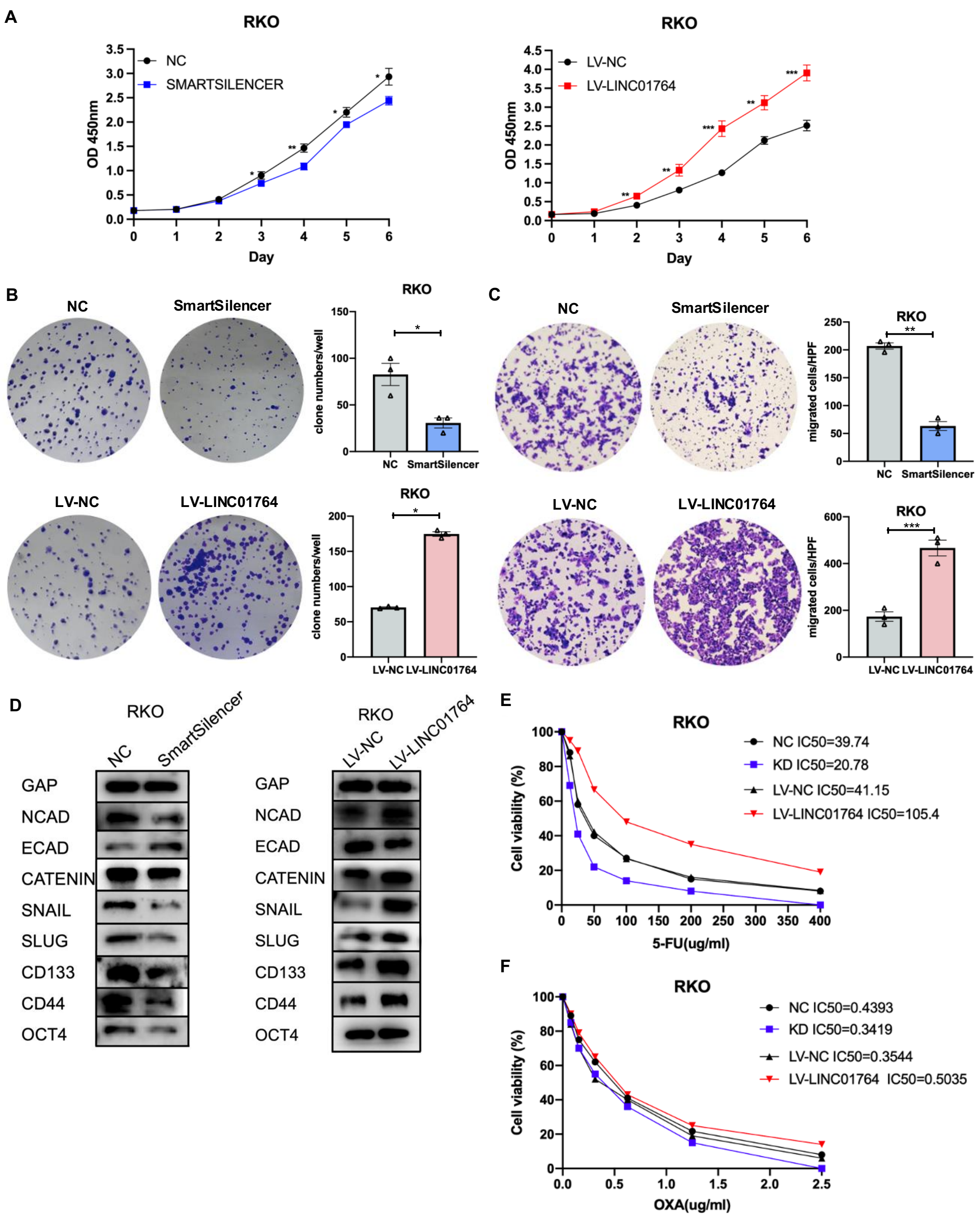

Fig. S1 LINC01764 facilitates the proliferation and migration of CRC cells and weakens its sensitivity to 5-FU in RKO cells

(A, B, C) Cell proliferation and migration were assessed by CCK-8 (A), colony formation (B) and transwell assays(C). Downregulation of LINC01764 significantly inhibited the proliferation and migration of RKO while upregulation of LINC01764 promoted proliferation and migration. (D) Western blot analysis was performed to assess the protein levels of N-cadherin, E-cadherin, β-catenin, Snail, Slug, CD133, CD44 and Oct4 after knocking down or overexpressing LINC01764

in RKO cells. (E, F) IC50 of 5-FU was significantly decreased when LINC01764 was downregulated while increased when LINC01764 was upregulated in RKO cells. *P < 0.05, **P < 0.01, ***P < 0.001.

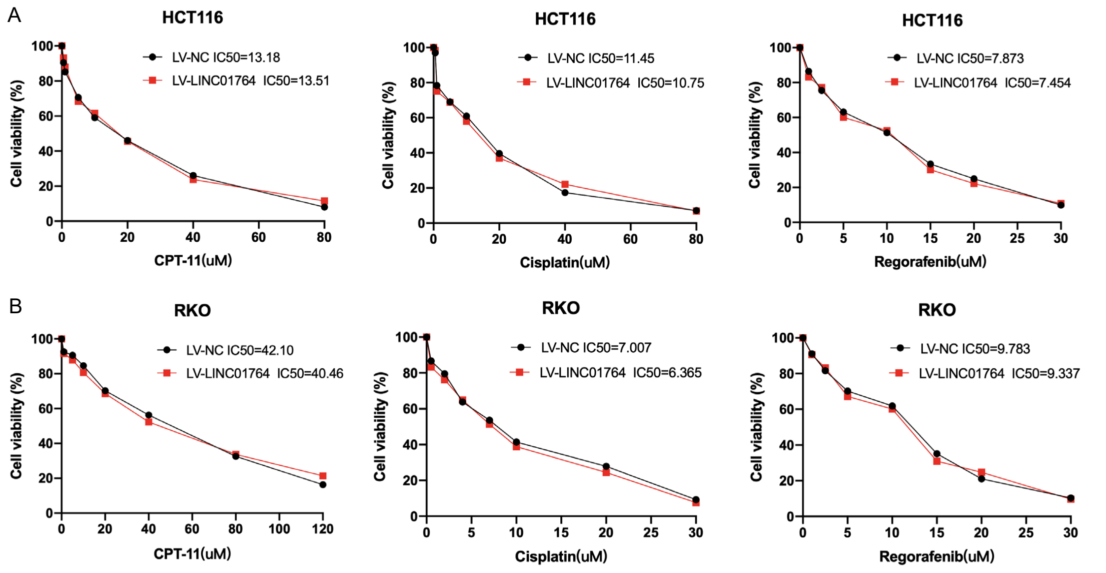

Fig. S2 LINC01764 does not affect the sensitivity of CRC cells to other chemotherapeutic and targeted drugs.

(A, B) IC50 of CPT-11, Cisplatin and Regorafenib were not significantly changed when LINC01764 was upregulated in HCT116(A) and RKO(B) cells. All data are shown as the mean ± SEM values.

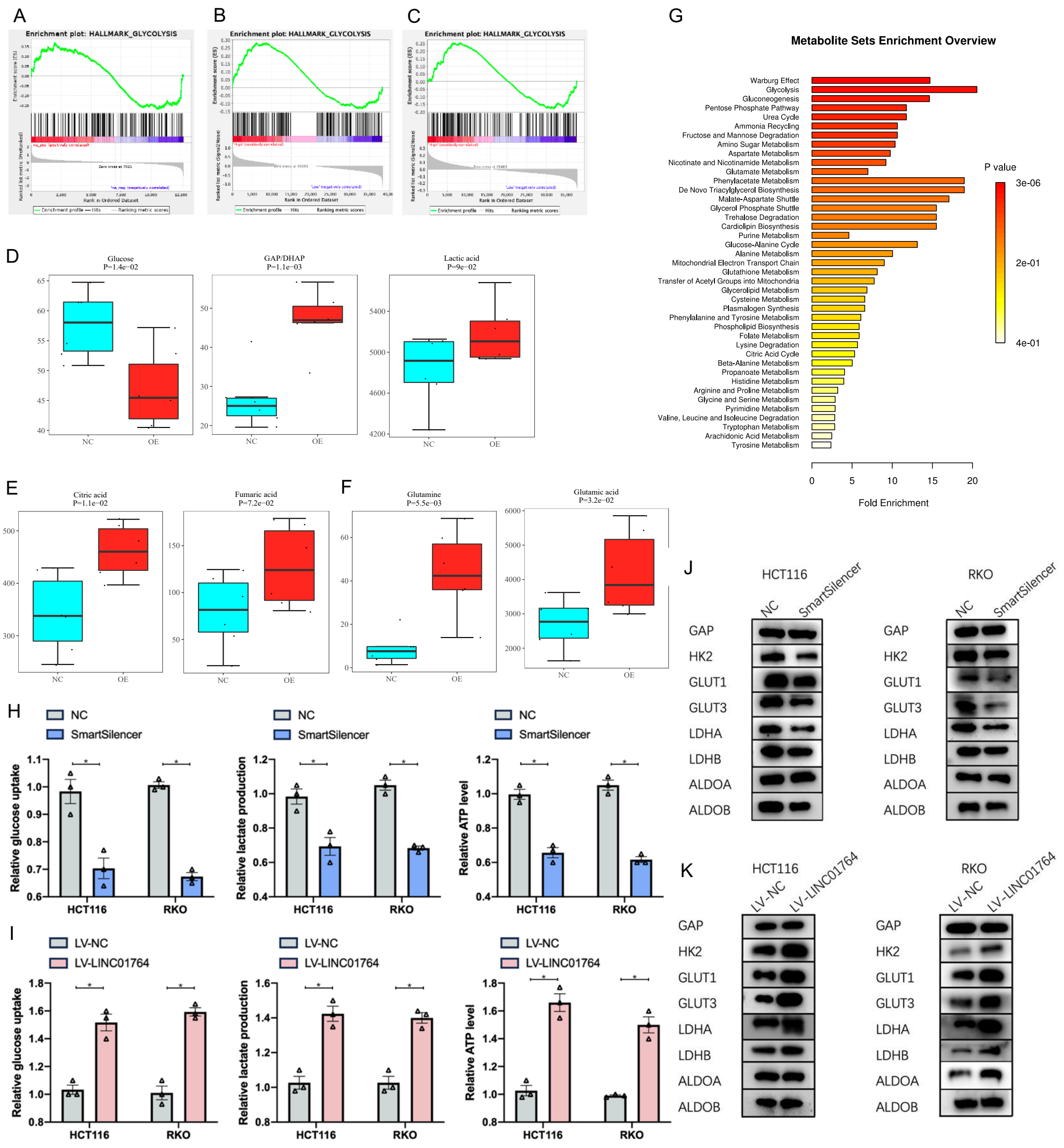

Fig. S3 LINC01764 promotes glucose metabolism.

(A-C) GSEA analysis of LINC01764 stable overexpression cells (A), FDUSCC dataset (B) and TCGA-COAD dataset (C) showed that upregulation of LINC01764 is correlated with activation of glycolysis pathway. (D-F) Boxplot of seven top differential metabolites. (G) Pathway enrichment analysis barplot using pathway-associated metabolite sets (SMPDB). (H, I) Measurements of glucose uptake, lactate production and ATP level in HCT116 and RKO cells after LINC01764 knocking down and overexpressing. (J, K) Western blot analysis was performed to assess the protein levels of HK2, GLUT1, GLUT3, LDHA, LDHB, ALDOA and ALDOB after knocking down or overexpressing LINC01764 in HCT116 and RKO cells, as indicated, GAPDH was used as the loading control. All data are shown as the mean ± SEM values. *P < 0.05.

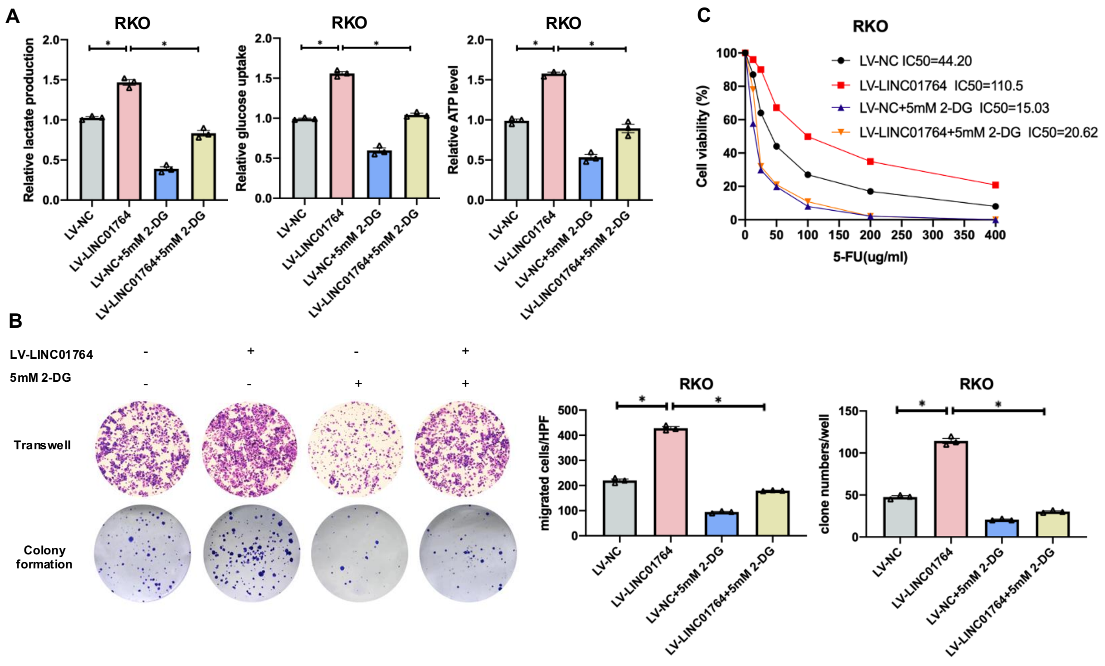

Fig. S4 LINC01764 performs its oncogenic functions by promoting glucose metabolism

(A) Measurements of glucose uptake, lactate production and ATP level in RKO after LINC01764 overexpressing and 2-DG treatment. (B-C) Cell proliferation, migration and 5-FU sensitivity of LINC01764 overexpression cells after 2-DG treatment were assessed by colony formation (B), Transwell assays (B), and IC50 determination (C). 2-DG treatment eliminated the promoted proliferation, migration and 5-FU resistance caused by upregulation of LINC01764 (B, C).

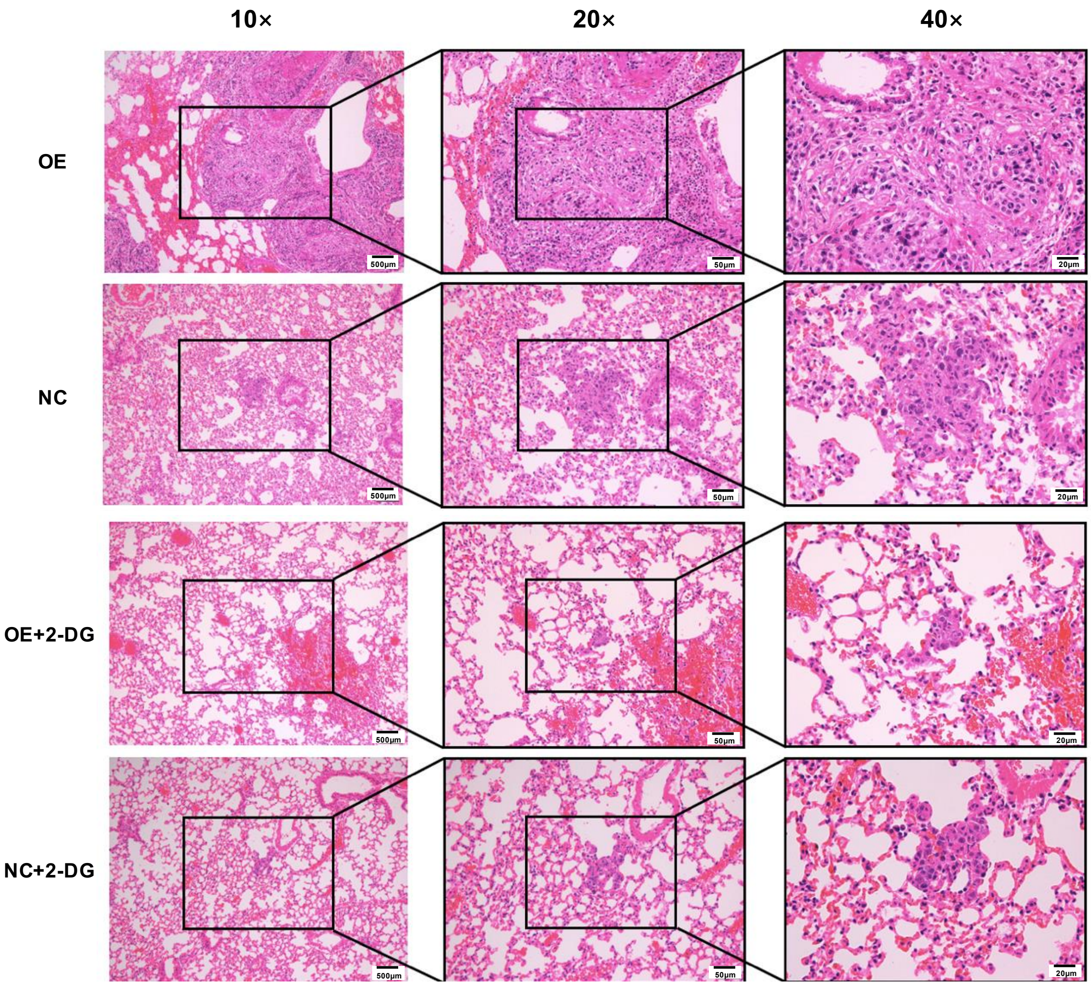

Fig. S5 (A) Representative HE staining images of lung metastasis.

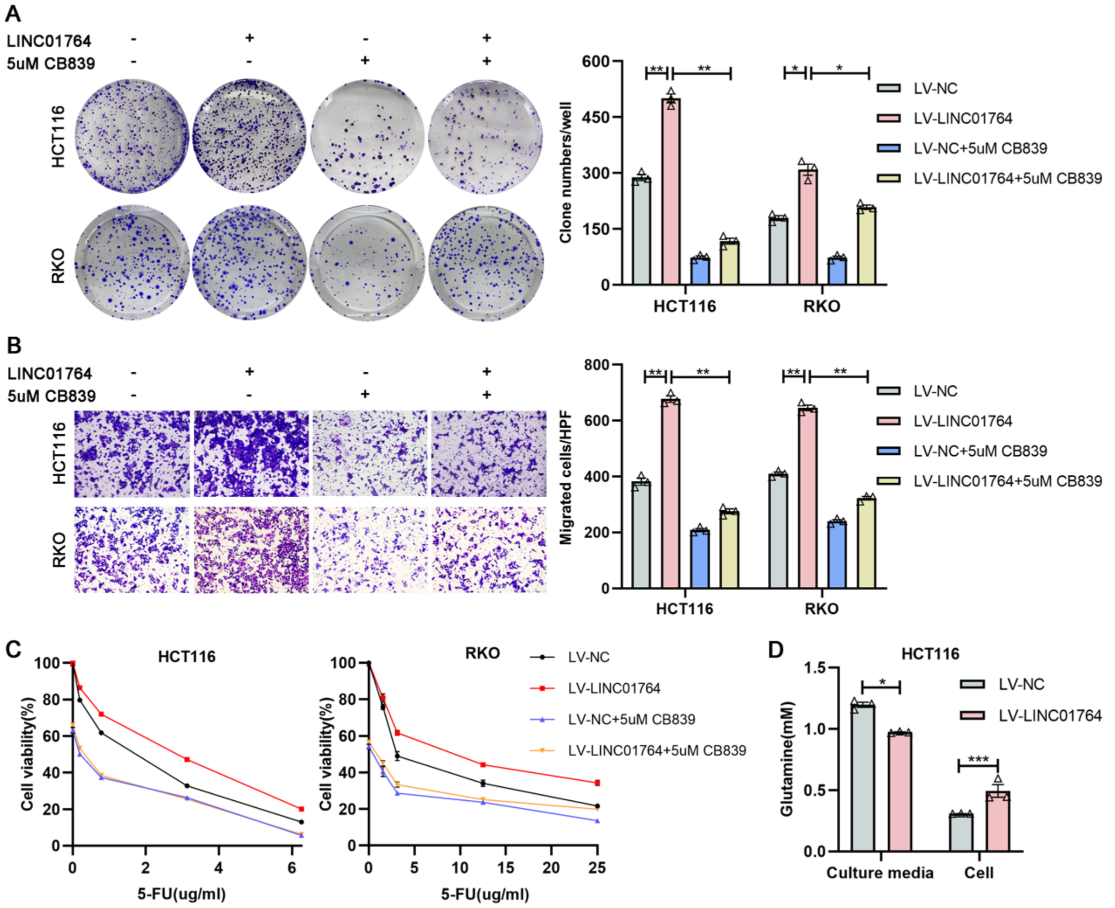

Fig. S6 LINC01764 promotes proliferation, migration and 5-FU resistance of CRC cells via regulating glutamine metabolism.

(A) Colony formation assay (left panel) were performed to evaluate the proliferation ability of CRC cells after LINC01764 overexpression or 5uM CB839 treatment, and the number of colonies was counted (right panel). (B) Transwell assay (left panel) were performed to evaluate the migration ability of CRC cells after LINC01764 overexpression or 5uM CB839 treatment, and the number of migrated cells was counted (right panel). (C) CCK-8 assay was performed to evaluate the sensitivity of CRC cells to 5-FU after LINC01764 overexpression or 5uM CB839. (D) After replacing the fresh complete medium for 48 hours, the medium and intracellular glutamine concentrations were measured in HCT116 cells overexpressing LINC01764 and control cells. All data are shown as the mean ± SEM values. *$P < 0.05$, **$P < 0.01$, ***$P < 0.001$, NS=No Significance

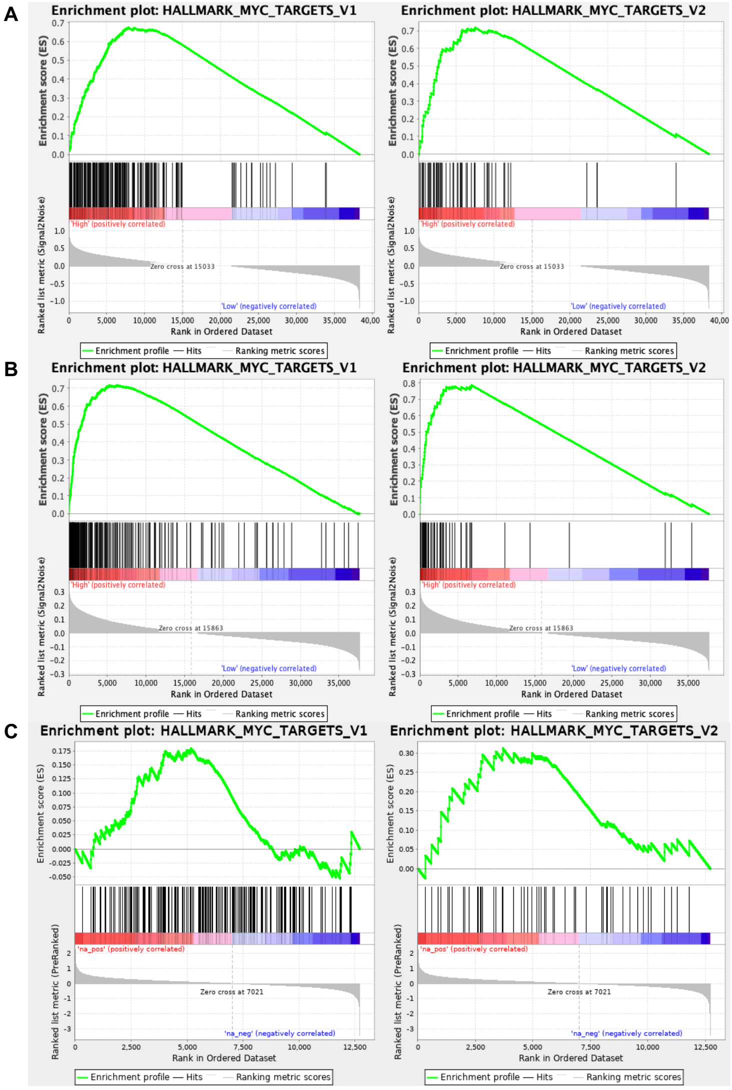

Fig. S7. Gene set expression analysis (GSEA) of datasets from FDUSCC, TCGA, and cell RNA-seq

GSEA demonstrated that a high LINC01764 expression correlates with activation of the MYC signaling pathway

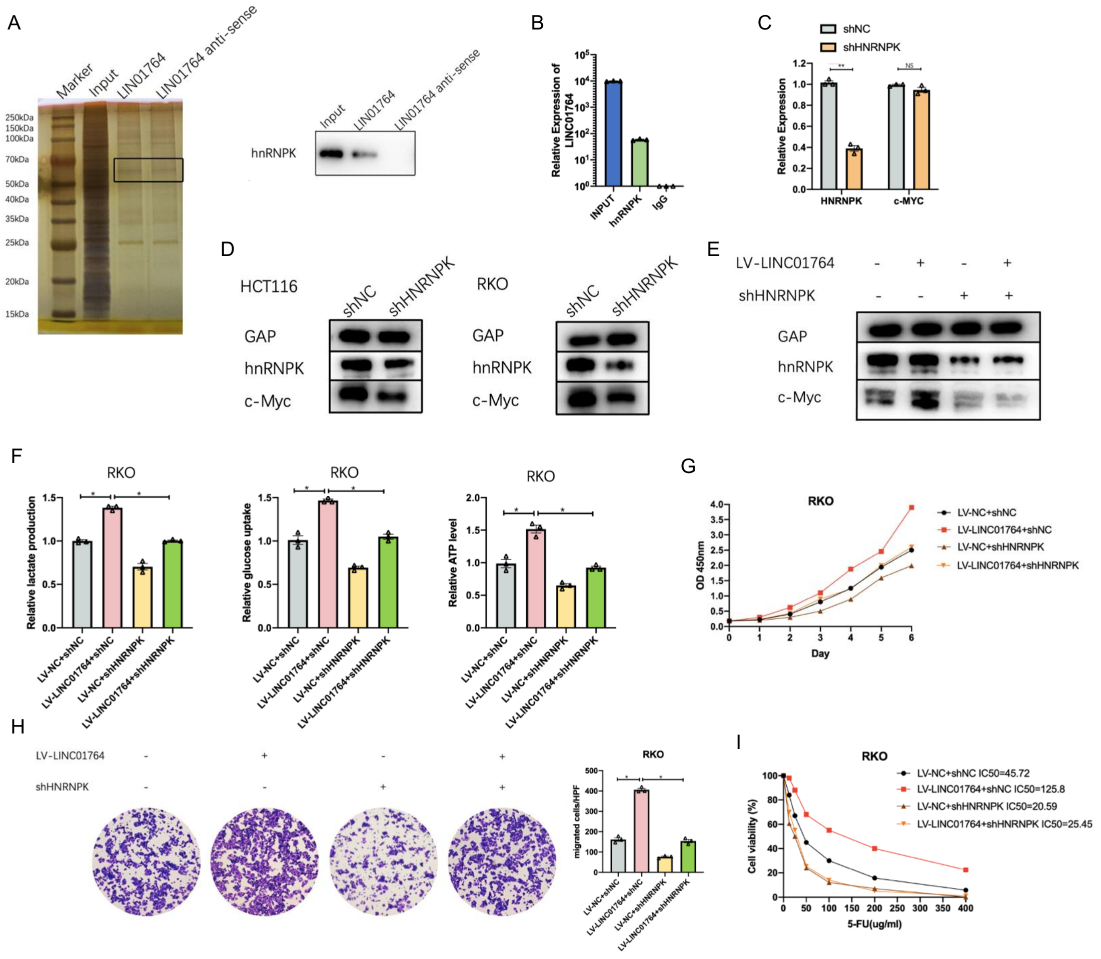

Fig. S8 LINC01764 regulates c-Myc protein expression and thus functions through specifically binding to hnRNPK.

(A) RNA pull-down was performed to find the specifically binding protein to LINC01764. (B) RIP assay was performed to confirm specific binding of LINC01764 and hnRNPK. (C) The expression of HNRNPK and c-MYC were analyzed by qRT-PCR after HNRNPK knocking down. (D) The expression of hnRNPK and c-Myc in HCT116 and RKO cells were analyzed by Western blot after HNRNPK knocking down. (E) Western blot analysis was performed to assess the protein levels of hnRNPK and c-Myc after co-transfection of LV-LINC01764 and shHNRNPK, as indicated, GAPDH was used as the loading control. (F) Measurements of glucose uptake, lactate production and ATP level in RKO cells after co-transfection of LV-LINC01764 and shHNRNPK. (G-I) Cell proliferation, migration and 5-FU sensitivity of RKO cells after co-transfection of LV-LINC01764 and shHNRNPK were assessed by CCK-8 (G), Transwell assays (H), and IC50 determination (I). Downregulation of HNRNPK eliminated the promoted proliferation, migration and 5-FU resistance caused by upregulation of LINC01764. All data are shown as the mean ± SEM values. *P < 0.05,

**P < 0.01.

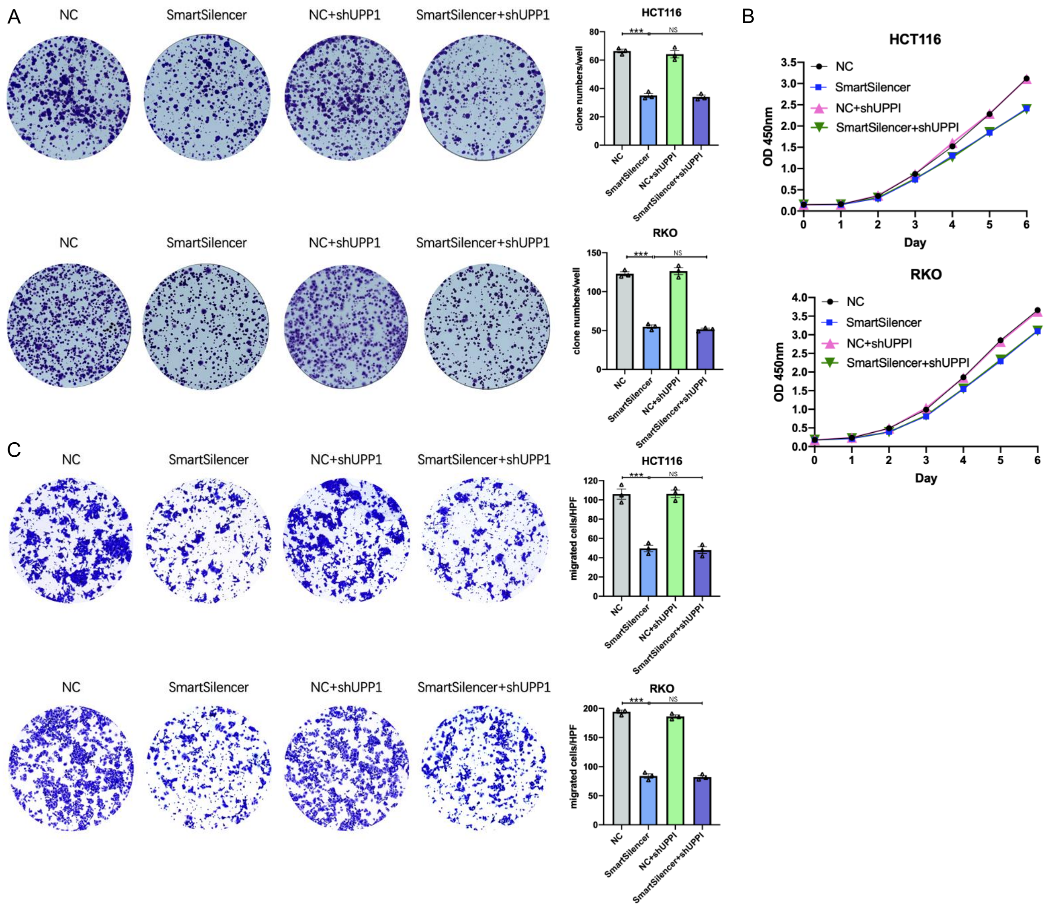

Fig. S9 LINC01764 does not depend on *UPP1* to promote the proliferation and migration of CRC cells.

(A, B) Cell proliferation was assessed by colony formation (A) and CCK-8 (B) after knocking down of LINC01764 and UPP1. (C) Cell migration was assessed by Transwell assays after knocking down of LINC01764 and UPP1. All data are shown as the mean ± SEM values. *P < 0.05, **P < 0.01.

Supplementary Table1

Association of LINC01764 expression detected by FISH in colon cancer tissues with clinicopathological characteristics.

	LINC01764 expression		
Parameter	High	Low	P- value
Age			0.6882
<68	9	7	
≥68	4	5	
Gender			
Male	9	7	0.6882
Female	4	5	
Gene mutation (KRAS,NRAS,BRAF)			
Yes	4	4	0.9999
No	7	6	
Missing	2	2	
Differentiation			
Well and Moderate	8	9	0.603
Poor	1	3	
Missing	4	0	
Tumor location			
Left -sided	10	11	0.593
Right-sided	3	1	

Supplementary Table2

Association of LINC01764 expression detected by PCR in colon cancer tissues with clinicopathological characteristics.

	LINC01764 expression		
Parameter	High	Low	P- value
Age			>0.9999
<68	6	7	
≥68	7	8	
Gender			>0.9999
Male	8	9	
Female	5	6	
Gene mutation (KRAS,NRAS,BRAF)			>0.9999
Yes	4	4	
No	5	7	
Missing	4	4	
Differentiation			>0.9999
Well and Moderate	2	3	
Poor	11	12	
Tumor location			
Left -sided	8	12	0.4097
Right-sided	5	3	
